# Mental health nurses' measured attitudes to people and practice: Systematic review of UK empirical research 2000–2019

**DOI:** 10.1111/jpm.12826

**Published:** 2022-02-23

**Authors:** Geoffrey L. Dickens, Mariyana Schoultz, Nutmeg Hallett

**Affiliations:** ^1^ Department of Nursing, Midwifery, and Health Northumbria University Newcastle upon Tyne UK; ^2^ School of Nursing College of Medical and Dental Sciences University of Birmingham Birmingham UK

**Keywords:** mental health, personality disorders, psychiatric nursing attitude, psychometrics, systematic review, violence

## Abstract

**What is known on the subject?:**

- Many studies have investigated the attitudes of mental health nurses towards a range of targets. These targets are person‐oriented (for example groups of people with a similar mental health diagnosis) or practice‐oriented (for example practices such as seclusion or restraint).
- It is thought that attitudes contribute to the practice of mental health nurses because research suggests attitudes have a role in shaping behaviour.

**What the paper adds to existing knowledge?:**

- To date, research about mental health nurses' attitudes has examined different attitudes in isolation from one another. By demonstrating a lack of connectedness across studies this paper highlights the need for new theory‐informed approaches to attitudinal research.
- By standardizing measurements across different studies this review demonstrates that the most negatively appraised attitudinal targets—indicated by large proportions of respondents who appraise negatively—concern people with diagnoses of borderline personality disorder, substance misuse, and acute mental health presentations.

**What are the implications for practice?:**

- Significant numbers of mental health nurses may have attitudes, especially towards people with borderline personality diagnoses and those who misuse substances, that may not be concordant with good practice.
- There is insufficient evidence about what the actual implications this has for practice because the body of relevant research lacks coherence, interconnectedness and a grounding in contemporary theoretical developments.
- Training programmes that focus on attitudinal change need to be more rigorously evaluated.

**Abstract:**

**Introduction**

Attitudes are considered integral to mental health nursing practice.

**Aims**

To comprehensively describe the (i) measured attitudes of UK mental health nurses towards people and practice; (ii) effectiveness of interventions to change attitudes; and (iii) relationships between their attitudes, other variables/constructs and practice.

**Methods**

Using systematic review methodology, multiple databases (CINAHL, Scopus, PsycINFO, Web of Science Core Collection, Google Scholar) were searched. Eligible studies involved measurement of UK‐based mental health nurses' attitudes with multi‐item scales. Studies were quality appraised, mean (SD) attitudinal data were standardized, and other results converted to standardized effect sizes.

**Results**

*N* = 42 studies were included. Negatively appraised attitudinal targets were people with a borderline personality disorder diagnosis, substance misuse, and acute mental health presentations. Educational interventions were associated with immediate increases in positive appraisals but sustainability was poorly evidenced. There was very limited study of attitude‐practice links.

**Discussion**

This review identifies priority attitudinal targets for action but also demonstrates that future work must consider the interconnectedness of attitudes and their relationship with practice.

**Implications for Practice**

Priority areas for consideration are attitudes to borderline personality disorder, substance misuse and mental health co‐morbidity. Addressing disparities between nurses' attitudes and those of service users is important. More robust research is required into the effectiveness of interventions to change attitudes and into attitude‐practice links.

## 1 INTRODUCTION

Over the last two decades, the attitudes of mental health nurses have continued to be the subject of a range of empirical research. Investigations into attitudes have fallen into two broad categories. First are studies of mental health nurses' attitudes towards the people for whom they provide care including their diagnoses and behaviours. Target attitudes in this category have included diagnostic categories like personality disorder (e.g., Bowers, Whittington, et al., 2006; Dickens et al., 2018) and severe mental illness (Chambers et al., 2010), and behaviours including aggression and self‐harm (Jansen et al., 2005; Patterson et al., 2007a,2007b). Second, studies have examined nurses' attitudes towards their own practices; these include containment measures (Bowers et al., 2007), self‐cutting management (Hosie & Dickens, 2018) and neuroleptic treatment (Harris et al., 2007). The volume of studies about mental health nurses' attitudes suggests that they are perceived to be of considerable importance. However, there has not to our knowledge been any overarching literature review which consolidates and integrates knowledge about attitudes in mental health nursing, nor any which develops theory by examining relationships between the array of attitudes studied or between them and other important variables.

## 2 BACKGROUND

### 2.1 Defining attitudes

“Attitude” is defined as "*n*. a relatively enduring and general evaluation of an object, person, group, issue, or concept on a dimension ranging from negative to positive. Attitudes provide summary evaluations of target objects (in this paper we use the term ‘targets’) and are often assumed to be derived from specific beliefs, emotions, and past behaviours [sic] associated with those objects" (American Psychological Association, 2022). In the field of social psychology, they are operationally understood to comprise cognitive (“I think that…”), emotional (“I feel that…”) or observable behavioural components (e.g., a rude or offhand manner) termed attitude content. The first two types of attitude are, from this perspective, associations in memory between an attitude object (the “thing” one is having an attitude toward) and an individual's personal evaluation of it (Maio et al., 2018). By “personal evaluation,” it is meant that the subjects' response is positive or negative, such as like or dislike, or potentially like *and* dislike. Under this definition, attitudes differ from other constructs which lack this evaluative component. Examples include beliefs, which are expressions of agreement or non‐agreement that a proposition is factually correct; perceptions, which are estimations of the extent, import or relevance of some construct; and knowledge, which is content from an organized body of facts that are generally held to be true and the response to which may be judged correct or incorrect.

### 2.2 Why attitudes are important

Given the widespread conduct of, and broad range of targets of, attitudinal research in mental health nursing, one could assume that attitudes are important per se. Indeed, some studies simply assert that links between attitudes and practice are axiomatic. For example, Acford and Davies (2019: p. 1177) state the common sense view that nurses report attitudes towards patients with a personality disorder diagnosis which “are likely to have a detrimental effect on the therapeutic engagement and care these individuals receive.” This may be true, but we consider it important to articulate the relevant theories about the underlying mechanisms behind such assertions.

Where they are voiced, studies of mental health nurses' attitudes invoke the importance of theory of planned behaviour (Ajzen, 1991) and theory related to stigma and its effects (Link, 1987). While addressing somewhat different issues—the first aims to understand why people behave the way they do and the second why some groups are stigmatized—both theories are central to attitudinal research because both view the underlying processes involved as amenable to intervention. Either or both theories are explicitly or implicitly appealed to by researchers when outlining the rationale for their investigations.

#### 2.2.1 Theory of planned behaviour

Ajzen (1991) posited that behaviour results largely from an individual's intention to behave in a certain way; however, that intention itself is influenced by key factors including her attitudes, perceived behavioural control (i.e., the extent to which she believes she has control over the behaviour), and subjective norms (i.e., the extent to which she perceives that others such as peers or managers also enact the behaviour). Further, these determinants of intended behaviour may also interact (e.g., attitudes partly influence perceived control and vice versa and so on). From this perspective, attitudes are important because they are linked with behaviour, opening up the possibility that behaviour itself might be susceptible to modification if they, and other contributing determinants such as knowledge, can be successfully addressed. As an illustration, Jansen et al. (2006) studied nurses' orientation to patient aggression using Ajzen's (1991) theory of planned behaviour as a theoretical framework. Theorizing that different approaches to the management of aggression across international boundaries might reflect the prevalent attitudes within nations, and thus partly determine the nature of actions required to successfully address behaviour, the authors studied those attitudes across five European countries including the UK. UK nurses were found to have significantly different attitudes to most other national groups in that they were more likely to view patient aggression as destructive, offensive and intrusive, and less likely to view it with tolerance. Of course, and as the authors allow, the study design used here does not allow for conclusions about the underlying reasons for the attitudinal differences found. Nevertheless, it demonstrates how the theory of planned behaviour has been used to justify and structure attitudinal investigation in mental health nurses.

#### 2.2.2 Stigma‐related theory

Stigma‐related theory, growing from the work of Goffman (1963) and furthered considerably in the specific instance of mental disorder by Link et al. (1987) posits that stigma is an attribute, behaviour or reputation which is socially discrediting in a particular way: it causes an individual to be mentally classified by others as an undesirable, rejected stereotype rather than as an accepted in‐group member (Goffman, 1963). Elaborating on this, Link and Phelan (2001) proposed that stigma arises from a perfect storm where (i) individuals differentiate and label human variations; (ii) prevailing cultural beliefs tie those with attributes labelled as adverse; (iii) labelled individuals are placed in distinct groups which serve to distinguish “them” from “us” (i.e., those with and without the “adverse” attribute); and (iv) individuals in “othered” groups suffer loss of status and discrimination resulting from the prior process. Because labels are essentially viewed as social constructs stigma can in theory be successfully challenged through techniques aimed at redefining people's understanding of the attributes held by people which have been negatively appraised. An example of the use of stigma theory in the UK mental health nursing literature is Markham's (2003) account of attitudes to people with a label of borderline personality disorder.

#### 2.2.3 Pragmatic justifications for mental health nurse‐related attitudinal research

More commonly, studies of mental health nurses' attitudes do not elaborate on underlying theoretical mechanisms; rather they simply point to existing empirical evidence of a link between attitudes and practice. In a study of attitudes towards suicidal behaviour, Anderson et al. (2000) point to evidence of an interaction between specific attitudes and the stigma of deliberate self‐harm which jeopardizes the effectiveness of professional interventions. Lamph et al. (2018) highlight an array of evidence that suggests a link between healthcare professionals' attitudes and the experience of patients with a diagnosis of borderline personality disorder (e.g., Bodner et al., 2015). In terms of nurses' attitudes to their own practice, in rationalizing their investigation of mental health nurses' physical healthcare‐related orientation, Robson and Haddad (2012) point to prior research linking attitudes to self‐efficacy and engagement in these practices (e.g., Howard & Gamble, 2011).

Studies of mental health nurses' attitudes have been justified by claims that the attitudinal target in question is an increasing policy priority (Baker et al., 2005); that in empirical studies mental health nurses have a demonstrated more negative appraisals than other professionals (Dickens et al., 2018); by a lack of knowledge about orientation towards a specific attitudinal target, for example, harm reduction approaches to self‐harm (James et al., 2017); and that it is important to change attitudes or at least study the effect of interventions on them (e.g., Lavelle et al., 2017). In summary, it is widely held that attitudes are important in mental health nursing.

### 2.3 A note on terminology

While “attitude” is not a uniformly used construct in psychology (see e.g., Maio et al., 2018) an underlying assumption of the theories and other justifications outlined above is that attitudes contribute to real‐world outcomes and are thus viewed as legitimate targets for interventions. The corollary of this is that, in addition to attitudes being inherently evaluative, there is also a *desired* direction such that attitudinal movement in the desired direction would, hypothetically, lead to changes in the associated real‐world outcomes also in the desired direction. From this perspective, it may make sense to refer to a specific attitude as “positive” if its' location on a two‐dimensional measurement scale is in the portion past the midpoint oriented towards the desired end of that scale and to use that term synonymously with a value judgement such as “good” or, in comparison with measured attitudes of some other entity, “better.” Similarly, appraisals towards the other scalar pole could be termed “negative” and used synonymously with evaluations like “bad” or “poorer.” However, while this might seem appropriate for some attitudes it is not so straightforward. For example, it may be reasonable to assume that measured attitudes that are more endorsing or supportive of people with a personality disorder diagnosis are the desired attitudes because we would expect them to be associated with real‐world outcomes like more respectful nurse‐initiated interactions or reduced stigma for the individual. However, for some attitudes, this may not be so clear cut. For example, in the case of attitudes to restrictive measures such as physical restraint, we are faced with two problems. The first, easily solved, is that presumably attitudes more endorsing of physical restraint as an intervention are not the desired attitudes. We could therefore simply reverse the terminology by identifying the less endorsing end of the scale as the desired attitude and label scores falling on the associated part of the scale as positive attitudes. This is undeniably confusing. There is potential for it to be even more so when the desired direction of the real‐world outcome is debatable. For example, attitudes to neuroleptic medication management have been conducted. There may not be an obvious desired direction of attitude. Do we require attitudes that endorse or reject their use? For these reasons, in this paper, we use language pertaining to attitudinal directionality with deliberate care. We use the terms “positive” and “negative” to indicate the direction of endorsement or rejection of the attitude construct under investigation rather than as a definitive indicator of the desired direction of that construct. We therefore eschew language indicating value judgements such as “good” or “bad” attitudes. When groups of individuals are compared then terms such as “more positive” are used to describe one group *relative* to another, and it should not be assumed that it indicates that either or indeed neither group are positive or negative in their appraisals overall.

### 2.4 Contribution of this paper

Current thinking in the study of attitudinal change suggests three important contextual areas: first, attitudes change in individuals across time as part of human development; second, attitudes exist in, and change resulting from, the context of social relationships, especially contact with powerful communicators; third, they exist in their own socio‐historical context and are subject to shaping by major events (Albarracin & Shavitt, 2018). We propose, therefore, that a viable contribution to the study of mental health nurses' attitudes can be made by examination of empirical studies from a specific temporal, geographical, and hence cultural, context. An attempt to identify and review the empirical literature on mental health nurses' attitudes in its entirety, and irrespective of context, would, in our view, be an unrealistically large task. While we do not rule out conducting increasingly broader reviews in future, we suggest that examination of mental health nurses' attitudes within a specific socio‐geographical‐historical context (UK 2000–2019) will synthesize existing knowledge such that it can enlighten our understanding of contemporary attitudes in our own context. It is likely to enlighten understanding of current contemporary priorities and highlight knowledge gaps for future research. The overall aim of this review was to systematically identify and appraise studies published 2000–2019 which measured the attitudes (outcome) of UK mental health nurses (population) towards any relevant attitudinal target (exposure or study focus) either cross‐sectionally or longitudinally (study type). Specific study objectives were to:

1. Identify what attitudinal targets mental health nurses appraise most positively and negatively and where the greatest polarizations lie.
2. Identify whether UK‐based mental health nurses' attitudes differ significantly from those of any other group studied (e.g., other professionals, public, service users, and non‐UK nurses).
3. Identify whether subgroups of UK‐based mental health nurses (e.g., gender, experience, and age) differ in their measured attitudes.
4. Identify whether measured attitudes of UK‐based mental health nurses change over time either in or out of the context of interventions to change attitudes.
5. Identify demonstrable associational or causal links between UK‐based mental health nurses different measured attitudes and between their attitudes, other constructs and practices.

## 3 METHODS

This review uses a systematic approach to identification and appraisal of mental health nurse‐related attitudinal research. For reasons discussed in the introduction, our review aims to address a specific socio‐geographical‐historical subset of all available research. We followed guidelines outlined in the Preferred Reporting Items for Systematic reviews and Meta‐analyses (PRISMA; Moher et al., 2009) to structure the study. There was no published protocol but the aims, search strategy, inclusion/exclusion criteria and quality assessment were established prior to study conduct.

### 3.1 Literature search strategy

We searched the CINAHL, Scopus (including Medline), PsycINFO and Web of Science Core Collection electronic databases in addition to Google Scholar. An example search is presented in Table 1. Two strategies were used. First, searching was undertaken on combinations of terms relevant to the population of interest (“mental health nurses,” “psychiatric nurses” and “mental health practitioners”) together with terms related to the outcome of interest, this being derived from combinations of terms related to “attitude” and a range of synonyms (e.g., “opinion,” “belief,” “perception” and “knowledge”) and to “measurement” and a range of synonyms (scale, measure, inventory, checklist, questionnaire and so on). The focus or exposure of interest was captured using a comprehensive set of terms related to mental health and related issues. In a second stage of the search, we entered the names (and abbreviations/acronyms) of all measurement tools identified in stage one (plus other measures identified from a broader Google Scholar search) in combination with the name of the tool's first author (see Appendix S2 for a complete list of tools searched). In addition, we followed up references in selected papers and undertook separate searches of the Table of Contents of a number of specialist mental health nursing journals. All searches were conducted in August 2020. All searches included geographical terms (United Kingdom, UK, England, Scotland, Wales, Northern Ireland, Great Britain, GB) as limiters and searches were limited to English language publications post‐1999. All search results were exported into EndNote X9 where duplicates were removed. The literature search strategy was devised by Author 1 and conducted independently by Author 1 (first two databases) and Author 2 (second two databases).

**TABLE 1:** Example search (CINAHL)

#	
1	((Mental Health or Psychiatr*) ADJ nurs*) OR ((Mental health OR Psychiatr*) ADJ practition*)
2	(Mental illness OR mental disorder OR mental health OR psychiatric illness OR psychiatric disorder OR schiz* OR personality disorder OR bipolar OR affective disorder OR mood disorder OR depression OR post traumatic OR PTSD OR anorexia OR bulimia OR eating disorder OR obsessive compulsive disorder OR psychosis OR psychotic OR substance use OR substance misuse OR substance abuse OR alcohol use OR alcohol misuse OR alcohol abuse OR addiction) OR (aetiolo* OR cause) OR (treatment OR care OR recovery OR ideology OR custodial OR aggression OR violence OR drug OR alcohol OR comorbid* OR stigma)
3	(Attitud* OR belief* OR opinion* OR percep* OR perceiv* OR perspect* OR concept* OR view* OR attribution* OR prejud* OR stigma OR knowledg* OR reaction*) ADJ (scale OR measure OR schedul* OR inventor* OR Questionnair* OR survey)
4	UK OR United Kingdom OR England OR Wales OR Scotland OR Northern Ireland OR Great Britain OR GB
5	PUBYEAR >1999
6	1 AND 2 AND 3 AND 4 AND 5

### 3.2 Inclusion and exclusion criteria

**FIGURE 1:**
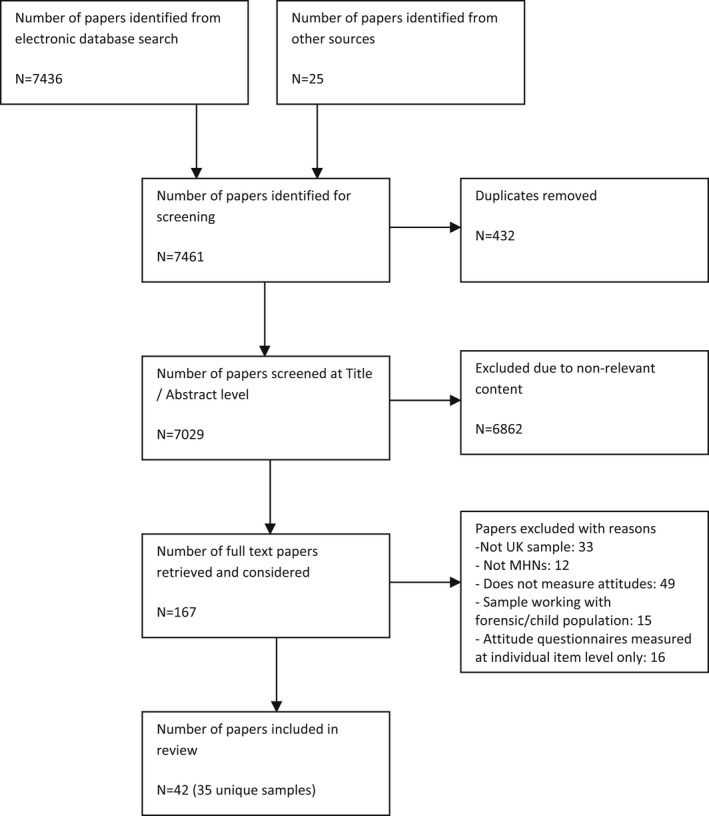
PRISMA Flow chart diagram

For inclusion, papers must have described an empirical study conducted with a sample comprising or including UK‐based registered mental health nurses and published in peer‐reviewed journals post‐1999. Studies of nursing students or solely of those working with forensic or older age/dementia populations were not included. Details of the application of inclusion criteria are presented in the PRISMA flow diagram (see Figure 1).

Studies were eligible for inclusion if they were designed in whole or part to measure attitudes. As an operational definition, we used that of the American Psychological Association (APA, 2022 see Introduction). We included studies using scales that self‐defined as “attitudinal” (e.g., Attitudes to Personality Disorder Questionnaire) but scales or subscales were not included where they were not self‐defined as attitudinal and were judged to measure knowledge, self‐efficacy or confidence, or any other dimension which we did not believe fit the APA definition. We operationalized “measure” as the use of a multi‐item (2+) scale with a summary score because there was no way to reasonably interpret studies which reported responses to questionnaires solely on an item‐by‐item basis. However, while we extracted information about the robustness of reported measures (e.g., internal reliability) and used it to inform our evidence synthesis, we did not exclude any study based on sub‐acceptable threshold scores.

### 3.3 Study quality/risk of bias

Studies were assessed against quality criteria adapted from two sources (Greenhalgh, 2006; University of York Centre for Reviews & Dissemination, 2008). Study quality and risk of bias were assessed by Author 1 with Author 3 independently conducting assessment of 50% of studies. Assessment considered categories related to study aims, sampling, questionnaire development and measurement, generalizability and funding (see Appendix S1). Discrepancies were discussed until consensus was achieved. Study quality assessment generated one or more overall quality rating for papers or groups of papers associated with a unique sample; this was because each attitudinal scale used with a sample was assessed on its own merits. All papers were included in the review regardless of quality.

### 3.4 Data extraction

We extracted information about study setting, sample (number and proportion of mental health nurses), the attitudinal construct investigated, the attitudinal and other tools used (subscale names/descriptors, number of items, and information about the internal reliability, test‐retest reliability or external validity of the tool based on the sample under investigation or cited in previous literature). Where possible, study results were extracted. Mean (*M*) and standard deviation (*SD*) scale/subscale scores were converted to a standardized *M* (*SD*) score and plotted in order to help gauge the sample's absolute level of attitudinal orientation, that is, the proximity of the mean to the scale midpoint and dispersal (*SD*) around the mean. As per our “note on terminology” (see above), we use the terms “positive” and “negative” to indicate level of endorsement of the scale items rather than as an indicator about the desired direction of the construct. There was one exception: the Self‐Harm Antipathy Scale (Patterson et al., 2007a,2007b) self‐evidently measures endorsement of statements which express feelings of aversion. In this instance, a pragmatic decision was made to simply reverse scores such that endorsement of such feelings was rated as a negative appraisal and rejection as positive appraisal. Where possible all inferential tests for difference or correlation were converted to a standardized effect size (Cohen's *d*) using an online converter (Wilson, 2001) in order to inform interpretation of the magnitude of differences or relationships between variables. We used the heuristic thresholds of *d *= 0.2 (small), 0.5 (moderate) and 0.8 (large) to interpret effect sizes. Data extraction was conducted by Author 1 while Author 3 independently extracted 50% of data to check for accuracy.

### 3.5 Synthesis of study findings

Despite a large number of relevant studies, there was excess heterogeneity in terms of samples and measures used to conduct meta‐analyses of study findings. Instead, we conducted a narrative review. We organized extracted information under top‐level headings regarding the orientation of attitudinal constructs investigated (person/behaviour or practice). Under each grouping, we organized evidence about the (i) absolute positivity or negativity of attitudes; (ii) positivity or negativity of attitudes relative to any other group, for example, mental health nurses vs. other professionals or service users or between UK and non‐UK samples; (iii) attitudinal differences within groups of mental health nurses or groups containing mental health nurses, for example, level of experience, gender; (iv) relationships between attitudinal constructs or between attitudinal and non‐attitudinal constructs; and (v) evidence of change in attitudinal constructs. Narrative syntheses were conducted bearing in mind the robustness of measurements based on their reliability and validity. Initial synthesis of the study findings was conducted by Author 1. These were checked and commented on by Authors 2 and 3 and were subsequently redrafted until consensus was achieved.

## 4 RESULTS

From the literature search strategy, we identified 42 papers describing studies of the attitudes of 35 unique samples comprising or including UK‐based mental health nurses published from 2000 to 2019 (see Table 2). Studies covered person‐oriented attitudinal targets including people in specific diagnostic categories (personality disorder *k *= 9; severe mental illness *k *= 4) and behaviours (aggression *k *= 10; self‐harm and suicidal behaviour *k *= 4, substance misuse *k *= 2); and practice‐oriented targets (attitude to containment or care *k *= 4; physical healthcare *k *= 2; medication management *k *= 3). A small number of studies included measures of more than one different attitudinal target (Bowers et al., 2008; Hosie & Dickens, 2018; Markham, 2003 *k *= 3 each). The most commonly used tools were the Personality Disorder Knowledge Attitudes and Skills Questionnaire (Bolton et al., 2010 cited in Shaw et al., 2012; *k *= 4), the Maslach Burnout Inventory depersonalization subscale (Maslach et al., 1997; *k *= 4), the Attitudes to Personality Disorder Questionnaire (Bowers & Allan, 2006; *k *= 3) and the Attitudes towards Containment Measures Questionnaire (Bowers et al., 2004; *k *= 3). Study quality assessment generated ratings of medium quality (scores of 5 to 8 of a possible 12) for 34 of 42 sample‐attitude tool combinations; three were rated low quality (scores of 4 or below) and five high quality (9 or above).

**TABLE 2:** Attitudes of mental health nurses (MHNs) study details

Study countries	Setting/population/sample	Design	Attitude target	Tool	Other measures (Bold = attitudinal)
Person‐oriented attitudes (i) Personality disorder (PD)					
[1] Acford and Davies (2019) UK	Psychiatric hospital, all wards 19 nursing staff *n* MHNs: 7	Cross‐sectional within a mixed methods study	Personality disorder	Personality Disorders Knowledge, Attitudes and Skills Questionnaire (PD‐KASQ; Bolton et al., 2010). Relevant subscale: “Emotional reaction”	PD knowledge Capability efficacy Therapeutic relationships
[2] Bowers, Brennan, et al. (2006) UK	Two acute admission psychiatric wards *N *= 58 staff *n* MHNs Unclear	Pre‐test ‐ 12‐mo post‐test trial. City Nurse supporting Safewards‐type model placed on wards	Personality disorder	Attitude to Personality Disorder Questionnaire (APDQ, Bowers & Allan, 2006): Relevant subscales: (i) enjoyment; (ii) security; (iii) acceptance; (iv) purpose; (v) enthusiasm	**Depersonalization** Conflict and containment Ward atmosphere Ward structure Satisfaction Staff‐patient interaction
[2b] Bowers, Whittington, et al. (2006), Bowers et al. (2008) UK	136 acute mental health wards *N*=1413 staff *n* MHNs: 973	Cross‐sectional survey	Personality disorder	APDQ (Bowers & Allan, 2006) Relevant subscales: (i) enjoyment; (ii) security; (iii) acceptance; (iv) purpose; (v)enthusiasm	**Containment measures** **Depersonalization** Self‐harm on ward Ward security and environment Ward Atmosphere ‐Leadership Team Climate Conflict and containment
[3] Bowers et al. (2015) James et al., (2017) UK	32 mental health wards 395 staff *n* MHNs: 239	RCT of Safewards intervention	Personality disorder	APDQ (Bowers & Allen, 2006) Relevant subscales: (i) enjoyment; (ii) security; (iii) acceptance; (iv) purpose; (v) enthusiasm	**Self‐harm antipathy** Ward atmosphere Physical health Mental health
[4] Davies et al. (2014) UK	1 mental health trust 162 staff *n* MHNs: Unclear	Pre‐test ‐ 3‐mo post‐test within mixed methods design	Personality disorder	PD‐KASQ (Bolton et al., 2010). Relevant subscale: “Emotional reaction”	PD knowledge Capability efficacy Course evaluation
[5]Dickens et al. (2018) UK	1 NHS Board 28 nursing staff *n* MHNs: 25	Pre‐test ‐ 3‐mo post‐test within mixed methods design	Borderline personality disorder (BPD)	Borderline Personality Disorder Cognitive Attitudes Inventory Relevant subscales: (i) treatment characteristics; (ii) perception of suicidal tendencies; (iii) antagonistic judgements Emotional Attitudes Inventory Relevant subscales: (i) negative emotions; (ii) experienced treatment difficulties (both Bodner et al., 2011, 2015)	BPD Knowledge
[5a] Ebrahim et al. (2016) UK	Mental health service staff enrolled on training course 196 staff MHNs: Unclear	Pre‐test ‐ 6‐mo post‐test of 3‐day KUF training within mixed methods design	Personality disorder	PD‐KASQ (Bolton et al., 2010) Relevant subscale: “Emotional reaction”	PD knowledge Capability efficacy Training evaluation
[6] Lamph et al (2017) UK	NHS Trusts, universities 80 mixed professionals *n* MHNS: Unclear	Pre‐test ‐ 3‐mo post‐test evaluation of e‐learning sessions	Personality disorder	PD‐KASQ (Bolton et al., 2010) Relevant subscale: “Emotional reaction”	PD knowledge Capability efficacy
[7] Markham (2003) UK	One NHS Trust's mental health inpatient wards 71 nursing staff *n* MHNs: 50	Repeated measures factorial	Borderline personality disorder	Modified Social Distance Scale (MSDS; Ingamells et al., 1996) Single scale Beliefs About Dangerousness (BAD; Link et al., 1987); Single scale Treatment optimism scale (TO; Dagnan et al., 1998); Single scale	Working experience
Person‐oriented attitudes (ii) Self‐harm and suicidality					
[8] Anderson et al. (2000) UK	One general hospital including medical and psychiatric units 33/59 staff including 10 MHNs *n* MHNs 10/33	Cross‐sectional survey	Suicidal behaviour	Suicide Opinion Questionnaire (SOQ; Domino et al. 1982). Relevant subscale: (i) cry for help/threats not real; (ii) moral evil	–
[9] Patterson et al. (2007a,2007b) UK	N=91 nurses and others in post‐registration education and 153 mental healthcare professionals *n* MHNs: Unclear	Cross‐sectional survey and pre‐test and up to 48‐mo post‐test course evaluation	Self‐harm	Self‐Harm Antipathy Scale (SHAS; Patterson et al., 2007a) Relevant subscales: (i) competence appraisal; (ii) care futility; (iii) client intent manipulation; (iv) acceptance and understanding; (v) rights and responsibilities; vi) needs function	–
Hosie and Dickens (2018) UK	Recruited through networks 175 MHNs, 40 service users *n* MHNs: 175	Cross‐sectional survey	Self‐cutting management Self‐harm	SHAS (Patterson et al., 2007a). Low internal reliability and new analysis resulted in 3‐subscales explaining 62.9% of variance: (i) perceived manipulative functionality; (ii) perceived positive functionality; (iii) freedom of choice	**Self‐cutting management** **Containment measures**
Person‐oriented attitudes (iii) Aggression					
[10] Jansen et al. (2005), Jansen et al. (2006) Seven European countries including UK	Psychiatric hospitals and education settings N = 1963 including *n*=153 from UK] *n* MHNs: Unclear	Cross‐sectional survey	Inpatient aggression	Attitudes Towards Aggression Scale (ATAS; Jansen et al., 2005, 2006). Relevant subscales: (i) offensive; (ii) communicative; (iii) destructive; (iv) protective; (v) intrusive	–
[11] Whittington (2002) UK	Community mental health trust *N*=36/100 MHNs invited n MHNs: 36	Cross‐sectional survey	Aggression in mental health settings	Perception of Aggression Scale tolerance subscale* (Jansen, 2000). 12‐item (8 neutral statements about patient aggression and 4 positive statements) *Author proposes that tolerance is an attitude as generally defined by psychologists	Burnout including Maslach Burnout Inventory **depersonalization** subscale Demographics,
Whittington and Higgins (2002) UK & China	Psychiatric hospitals in UK and China *N*=108 Chinese and 28 UK nurses *n* MHNs: 28 (UK)	Cross‐sectional survey	Aggression	Perception of Aggression Scale (POAS; Jansen, 1997) Tolerance subscale (see above) The four positive items of the 12‐item scale here are totalled and analysed separately	Burnout personal accomplishment Demographics,
Person‐oriented attitudes (iv) Severe mental illness					
[12] Bradshaw et al., 2007	MHNs in post‐registration education *n* MHNS: 23	Quasi‐experimental. Pre‐ and 9‐mo post‐educational course	Schizophrenia	13‐item attitude subscale of an attitude and assumptions questionnaire about schizophrenia and related family work. Single scale.	Knowledge‐based multiple choice questions
[13] Guise et al. (2010) UK	3 psychiatric hospitals, *N *= 148 registered nurses invited (54.7% response) *n* MHNs: 81	Cross‐sectional survey	Mental illness	Community Attitudes towards the Mentally Ill (CAMI; Taylor & Dear, 1981). 40‐items. Relevant subscales: (i) authoritarianism; (ii) benevolence; (iii) social restrictiveness; (iv) community mental health ideology	None
Morris et al., 2011 Six European countries including UK	Settings unclear *N*=850/1242 registered nurses working in MH settings invited from 6 countries *n* MHNs: 48 (UK)	Cross‐sectional survey	Mental illness	CAMI (Taylor & Dear, 1981). Relevant subscales: (i) authoritarianism; (ii) benevolence; (iii) social restrictiveness; (iv) community mental health ideology	None
Person‐oriented attitudes (v) Substance misuse					
[14] Munro et al. (2007) UK	NHS mental health service Adult MH and addiction services 49 nurses n MHNs: 49	RCT of 4‐day staff training	Co‐morbid substance use and mental health problems	Co‐Morbidity Problems Perceptions Questionnaire (CMPPQ) adapted from the ‘well validated’ Alcohol Problems Perceptions Questionnaire (AAPPQ; Cartwright, 1980). 34‐items. Single scale. "a low total score represents a positive therapeutic attitude and a high total score represents a more negative therapeutic attitude" (Watson et al., 2003).	True/False Knowledge questionnaire
[15] Richmond & Foster UK	Acute hospital & community settings *N*=56 mental health practitioners *n* MHNs: Unclear	Cross‐sectional survey	Substance abuse	Substance Abuse Attitude Survey (Chappel et al., 1985). 50‐items. Five subscales: (i) treatment intervention orientation; (ii) treatment optimism; (iii) permissiveness; (iv) non‐moralism; (v) non‐stereotyping	Demographics
Person‐oriented attitudes (vi) Depersonalized reaction to service users					
Hannigan et al. (2000) UK	All NHS Trusts in Wales 301 (50% response rate) qualified CMHNs *n* MHNs: 301	Cross‐sectional survey	Negative attitudes to service users	Maslach Burnout Inventory (MBI; Maslach et al., 1986). Relevant subscale: 5‐item Depersonalization subscale capturing ‘development of cold negative attitudes towards people who use services’	MBI Emotional Exhaustion and Personal Accomplishment subscales Demographics Self‐esteem) Stress Coping General health
Laker et al. (2019)	Eight acute inpatient wards *N*=125 ward‐based staff *n* MHNs: 81	Part of an RCT for nurse‐led therapeutic interventions to improve ward climate	Negative attitudes to service users	MBI (Maslach et al., 1996). Relevant subscale: 5‐item Depersonalization subscale	Staff perceptions of barriers to change Demographics
Person‐oriented attitudes (vii) Acute mental health					
[16] Baker et al. (2005) Munro & Baker (2009) UK	Five acute specialist acute mental healthcare trusts *N*=140 staff *n* MHNs 94	Cross‐sectional survey, scale development	Acute mental health care	Attitudes Towards Acute Mental Health Scale (ATAMHS; Baker et al., 2005). 33‐items. Relevant subscales: (i) care or control; (ii) semantic differentials; (iii) therapeutic perspective; (iv) hard to help; (v) positive attitudes	None
Mistral et al. (2002) UK	One psychiatric “high care” inpatient unit *N*=36 staff *n* MHNs: 14	Pre‐test post‐test evaluation of intervention (regular staff meetings and use of global outcomes measure)	High care patients	The Attitude Measure (Mistral et al., 2002). Based on the AAPPQ (Cartwright, 1980)*. Relevant subscales: (i) feelings of skill and knowledge adequacy; (ii) self‐esteem in this work; (iii) willingness to work with these patients; (iv) satisfaction; (v) a right to work with these patients; (vi) role support; and (vii) general self‐esteem. *"a measure of an overall therapeutic attitude towards the alcoholic client" (Cartwright, 1980)	Ward atmosphere
Practice‐oriented attitudes (i) Containment measures					
[17] Bowers, Whittington, et al. (2006), Bowers et al. (2008) Whittington et al. (2009) UK	136 acute mental health wards 1226 staff (68% nurses) and 1361 patients *n* MHNs: 834	Cross‐sectional survey	Containment measures	Attitudes towards Containment Measures Questionnaire (ACMQ; Bowers et al., 2004). Relevant subscales relate to 11 containment measures: (i) PRN medication; (ii) compulsory intramuscular medication; (iii) physical restraint; (iv) intermittent observation; (v) constant observation; (vi) time out, (vii) PICU transfer; (viii) locked door seclusion; (ix) open‐area seclusion; (x) net bed; (xi) mechanical restraint. Each accompanied by an illustration and brief description. Each measure rated for effectiveness, acceptability, respectfulness, safety for patients, safety for staff, willingness to undergo/use	**Containment measures** **Depersonalization** Self‐harm on ward Ward security and environment Ward Atmosphere ‐Leadership Team Climate Conflict and containment
Bowers et al., 2010 UK	136 MH wards 638, 393 and 168 Patients, staff and visitors. *n* MHNs 197	Cross‐sectional survey	Attitudes to Locked Doors on acute mental health wards	ACMQ (Bowers et al., 2004)‐ paralleling ‘locked doors’ item	Effects of locked doors questionnaire
[19] Pettit et al. (2016) UK	Eight acute psychiatric inpatient hospitals *N*=206 staff *n* MHNs: 130	Cross‐sectional survey	Containment	ACMQ Version 2.0. Differs from ACMQ (Bowers et al., 2004) in that respondents’ rate only a single “acceptability” score	Progression of aggression Demographics Ward type and facilities
Practice‐oriented attitudes: (ii) suicide prevention					
[20] Sandford et al. (2020) UK	One NHS Trust 1012/ c. 4000 staff including 292 clinical mental health staff *n* MHNs: Unclear	Cross‐sectional survey	Suicide prevention	Attitudes to Suicide Prevention Scale (Herron et al., 2001). Single scale. 14‐items	Demographics
Practice‐oriented attitudes (iii) Self‐cutting management					
[18] Hosie and Dickens (2018) UK	Recruited through networks 175 MHNs, 40 service users n MHNs: 175	Cross‐sectional survey	Self‐cutting management Self‐harm	Attitudes to Self‐cutting Management scalE (ASc‐ME; Hosie & Dickens, 2018). 17 management techniques (i) seclusion; (ii) informing other staff; (iii) providing sterile razors; (iv) intermittent observations; (v) constant observations; (vi) physical restraint; (vii) give wound advice; (viii) passive distraction; (ix) provide first aid kit; (x) care planning; (xi) PRN with consent; (xii) active distraction; (xiii) refuse medical care; (xiv) forced intramuscular medication; (xv) therapeutic interventions; (xvi) inappropriate medical treatment; (xvii) remain present during cutting event. All rated (for effectiveness, acceptability, respectfulness, safety for staff, safety for patients, and preparedness to use/be subject to). ACMQ (Bowers et al., 2004) ASc‐ME‐paralleling items only	**Self‐harm antipathy** **Containment measures**
Practice‐oriented attitudes (iii) Psychotropic medication					
[21] Harris et al. (2007) UK	32 Trusts 238 multidisciplinary staff n MHNs: 202 (across study stages	Cross‐sectional surveys	Maintenance neuroleptic treatment	Staff Attitude to Neuroleptic Treatment Inventory (SANTI; Harris et al., 2007). 25‐items. Relevant subscales: (i) attitude; (ii) perception of skills	None
[22] Patel et al., 2005 UK [Further analysis in Patel et al. (2008)]	70/105 CPNs attending an academic meeting n MHNs: 70 plus 98 Hong Kong nurses	Cross‐sectional survey	Depot medication	Purpose designed questionnaire, 34‐items. Relevant subscales: (i) patient‐centred attitudes, (ii) non‐patient‐centred attitudes	None
Patel et al. (2009) UK	2 Trusts *N*=201 MHNs and psychiatrists *n* MHNs: 119	Cross‐sectional survey	MHN prescribing of psychotropic medication	Purpose designed. 65‐items. Relevant subscales: (i) General beliefs; (ii) Impact; (iii) uses	None
Practice‐oriented attitudes (iv) Physical health					
[23] Lavelle et al. (2017) UK	Two psychiatric triage units 53 staff n MHNs: 36	Pre‐test post‐test evaluation of medical emergency training within mixed methods design	Management of medical emergency in mental health settings	Purpose designed 4‐item self‐report attitude scale	Knowledge Confidence Incident reports
[24] Robson and Haddad (2012) UK [Supple‐mentary analysis in Robson et al., 2013]	585/1130 (52%) mental health nurses (397 inpatient and 171 community) n MHNs: 585	Cross‐sectional survey	Physical health care of people with mental disorder	Physical Healthcare Attitudes Scale for mental health nurses (PHASe; Robson & Haddad, 2012). 28 items. Relevant subscales: (i) attitudes to involvement in physical healthcare; (ii) barriers to physical healthcare delivery; (iii) attitudes to smoking	Demographics/ professional information Involvement in physical healthcare
Practice‐oriented attitudes (v) Miscellaneous					
Georgieva et al (2019) 11 Countries including UK	From researchers' networks 2616 mental health practitioners 102 in UK including nurses (20% nurses across whole sample) n MHNs: Unclear	Cross‐sectional survey	Mental health legislation	Mental Health Legislation Attitudes Scale, nine items. Single scale.	None
[25] Rogers et al. (2019) UK	Mental health trust 104 practitioners n MHNs: Unclear	Cross‐sectional survey	Spirituality	Modified spirituality in education attitudes questionnaire (Prentis et al., 2014). 13‐items. Relevant subscales: (i) Spirituality in everyday life; (ii) spirituality in practice	Views of spiritually competent practice Integration of spirituality in clinical education Distinctiveness of religion and spirituality
[26] Wood et al. (2007) UK	Mental Health trust 211 staff n MHNs: 160 (remaining participants are student MHNs)	Cross‐sectional survey	Electroconvulsive Therapy (ECT)	ECT attitude scale (Wood et al., 2007) 24‐items. Single scale	ECT knowledge Demographics Contact with patients having ECT

Note

**FIGURE 2:**
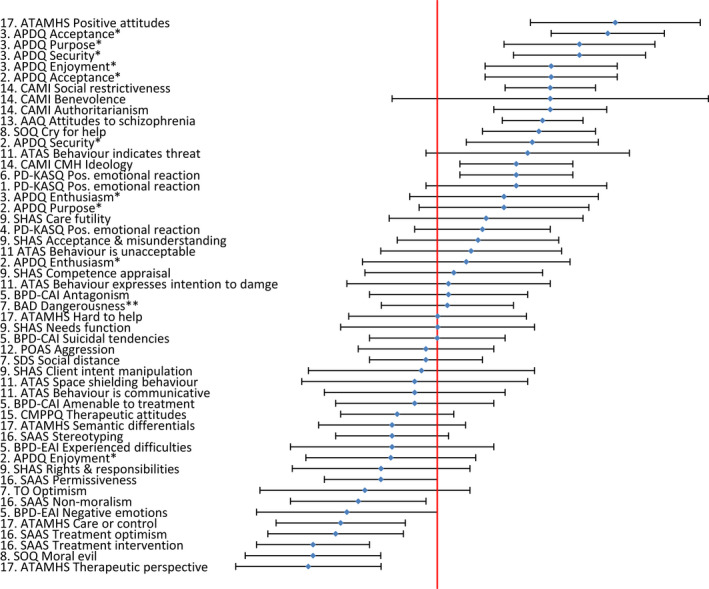
Person‐directed attitudes ([number] represents relevant study see Table 2, vertical line represents the standardized scale midpoint, diamond represents the standardized scale mean, and horizontal “error bars” represent standardized scale 1 standard deviation)

**FIGURE 3:**
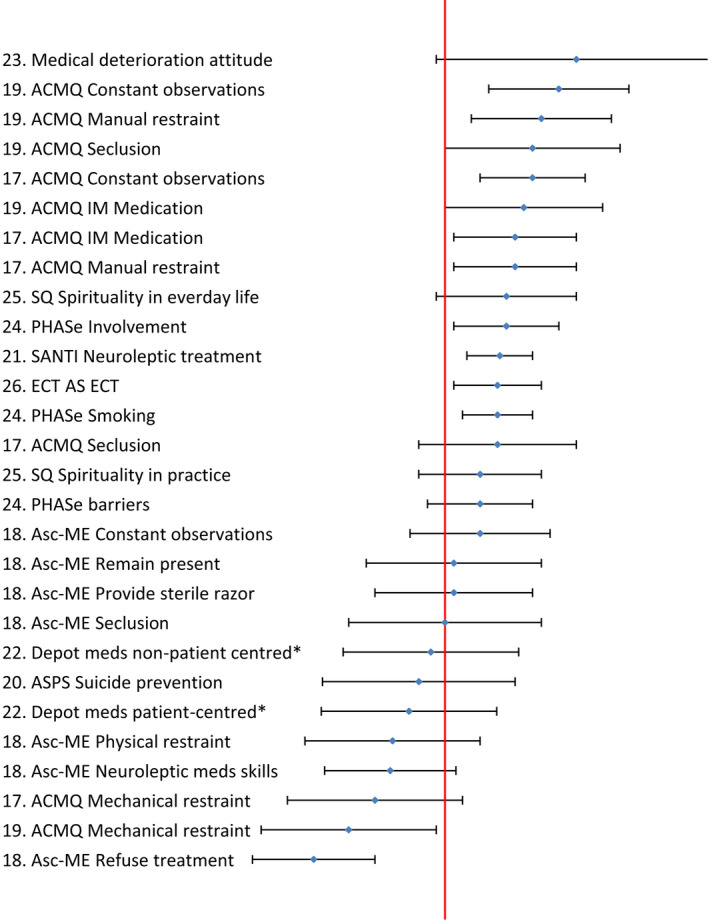
Practice‐directed attitudes ([number] represents study see Table 2, vertical line represents the standardized scale midpoint, diamond represents the standardized scale mean, and horizontal “error bars” represent standardized scale 1 standard deviation)

[Number] indicates number assigned to study for attitude scale scores extracted, standardized and included in Figures 2 and 3.

### 4.1 Person‐oriented attitudes

Person‐oriented attitudes were measured in 24 samples (see Table 2). For these studies, an acceptable or near acceptable internal reliability coefficient was usually reported for the sample or cited from prior research.

#### 4.1.1 Objective 1: Positive, negative and polarized appraisals

Fifty‐one standardized *M* (*SD*) ratings were extracted from 16 studies (see Figure 2); *n *= 10 standardized *M* (*SD*) ratings lay entirely to the right of the midpoint of their scale suggesting—given assumptions of normal distribution—that the majority of respondents positively endorsed attitudinal statements on that subscale. Contrastingly, negative appraisals (judged by standardized *M* (*SD*) ratings to the left of scale midpoint) were reported in relation to seven measures. All other standardized standard deviations were dispersed across the midpoint to some extent. Means with the widest *SD*s, suggesting more polarized appraisals, were as follows: the extent to which self‐harm represents an intention of manipulation (Patterson et al., 2007a,2007b), the level of optimism reported by mental health nurses about people with a diagnosis of borderline personality disorder (Markham, 2003), and the amount of enthusiasm reported for working with people with personality disorder (Bowers et al., 2006).

#### 4.1.2 Objective 2: Attitudinal differences between UK mental health nurses' and other groups

Other findings with contextual quality and effect size summaries are presented in Table 3. Evidence of differences between UK mental health nurses' attitudes and other groups were sparse but of interest. Markham (2003) manipulated case vignettes to examine differential responses of mental health nurses and healthcare assistants on attitudinal measures of desire for social distance, beliefs about dangerousness and treatment optimism. Vignettes presented identical scenarios but with diagnostic categories of borderline personality disorder, schizophrenia and depression attached serially across three presentation iterations. While mental health nurses did not rate measures of desire for social distance, beliefs about dangerousness or treatment optimism significantly differently from healthcare assistants, there was a significant interaction effect such that, for all three measures, mental health nurses rated the borderline personality disorder‐vignette significantly more negatively than both depression and schizophrenia instances while healthcare assistants did not. Effect sizes for mental health nurses' differential ratings were large for all three measures. Bradshaw et al. (2007) found no baseline differences in a measure of attitudes to schizophrenia among groups of mental health nurses and other students on university courses in their interventional study. Whittington and Higgins (2002) found more positive appraisals of statements relating to tolerance of aggression among UK mental health nurses than a comparison group from China. Patterson et al. (2007a,2007b) found significantly less endorsement of statements relating to their antipathy (large effect size) to patients' self‐harming behaviour among a sample including mental health nurses than in a comparison sample of general health qualified practitioners.

**TABLE 3:** Summary of findings regarding attitude measures

	Attitude target	Within/between group difference	Change over time	Relationship with other measures
Person‐oriented attitudes	Personality disorder	–	Short‐term positive change following educational intervention (Acford & Davies, 2019 [M]; Davies et al., 2014 [L]; Ebrahim et al., 2016 [M]; Lamph et al., 2018 [M]), ES+++ for “emotional reaction.” Evidence for sustained change hampered by low retention No change Bowers et al., 2006 [M] 2015 [M] pre‐ and post‐ Safewards‐type interventions	Bowers, Whittington, et al. (2006) [M] Data‐derived factors relating to ward conflict associated with APDQ low “security”; security provision with low APDQ “acceptance”; and observation with low APDQ “purpose” and “enthusiasm” (ES not calculable)
	Borderline personality disorder	MHNs rated BPD vignette more poorly than a schizophrenia vignette on social distance, dangerousness and treatment optimism while healthcare assistants did not ES+++ (Markham, 2003 [M])	Dickens et al., 2018; [M] Short‐term positive change following educational intervention. ES +++ for “treatment characteristics,” “perception of suicidal tendencies” and “negative attitudes.” Evidence for sustained change hampered by low retention	Hosie and Dickens (2018) [H]correlation between supportive attitude to harm reduction techniques and more positive self‐harm antipathy rating
	Suicidal behaviour/ self‐harm	MHNs less antipathy to self‐harm than those with general nursing‐only qualification ES+++ [M]	Self‐harm antipathy positive changes in short term (Patterson et al., 2007b). ES++ [M]. Evidence for sustained change hampered by low retention	Hosie and Dickens (2018) [H]. See cell above
	Severe mental illness	MHNs vs. other university students =no difference. Bradshaw et al. (2007) [M]	MHNs no significant change over time. Bradshaw et al. (2007) [M]	–
	Aggression	UK MHNs >tolerant than Chinese Whittington and Higgins (2002) [L] UK respondents judge aggression more negatively on dimensions related to offensiveness, destructiveness protectiveness and intrusiveness compared with Europeans ES++ (Jansen et al., 2006 [M])	–	MBI depersonalization subscale was negatively correlated with tolerance of aggression (ES+++ Whittington (2002) [M]; though this relationship was not detected in a different sample (Whittington & Higgins, 2002) [L]
	Depersonalized behaviour to patients	MHNs with non‐elderly caseloads, no job security and male staff more depersonalized ES++. No difference for those with supportive vs. unsupportive manager (Hannigan et al., 2000) [H]	–	MBI depersonalization subscalḥression tolerance: See above cell MBI depersonalization correlated with general health (ES+), self‐esteem (ES++), stress (ES++) Hannigan et al. (2000) [H} MBI depersonalization linked to reduced self‐reported efficacy to effect change ES non‐calculable Laker et al. (2019 [H])
Practice‐oriented attitudes	Self‐harm management	Hosie and Dickens (2018) [H] Nurses rated nine of seventeen techniques more favourably than prior users of services, mostly the least restrictive ones but also seclusion and physical restraint ES+to ES+++	–	Approval ratings for overlapping management techniques for self‐cutting and containment measures in general were correlated ES++ to ES++) Hosie & Dickens, 2018 [H]
	Containment measures	Whittington et al. (2009) [M]: Staff respondents appraised all methods of containment more positively than service users except open‐area seclusion, mechanical restraint and net bed (ES+to ES+++) Bowers, Whittington, et al. (2006) [M]: Male respondents appraised all techniques more favourably than females except time out, PICU, and IM medication [M] ES+. Younger staff more approving of mechanical restraint and net beds ES+. Staff who had used specific measures more approving of them (ES not calculable). Differences by UK geographic region ES+to ES++	–	Attitudes to containment measures not a significant independent predictor of all self‐harm or moderate self‐harm. A data‐derived factor comprising variables related to drug/alcohol use and absconding was associated with greater acceptability and safety for patients of containment measures (Bowers, Whittington, et al., 2006 [M])
	Neuroleptic treatment	Community MHNs more positive attitudes to neuroleptic medications than ward‐based MHNs (ES+++) and support staff ((ES++) Harris et al. (2007 [M]). MHNs more positive attitudes about MHN prescribing than psychiatrists Patel et al., 2009 [M] ES non‐calculable	–	–
	Physical healthcare	Additional general nursing qualification associated with “confidence in physical healthcare” (ES++) and “smoking cessation attitude” (ES+). Past 5‐y training in physical healthcare associated with overall attitudes to physical healthcare (ES++) Robson and Haddad (2012 [H])	–	–
	Physical deterioration	–	Improved medical emergency‐related attitudes immediately following simulation‐based training [ES+] Lavelle et al., 2017 [M]	
	Mental health legislation	Only respondents from the UK and Denmark rated >70% positivity towards their country's relevant mental health law (Georgieva et al., 2020 [M])	–	–

Abbreviations: –, no relevant findings. MHN =Mental health nurse; [L] [M] [H], Low, medium, high study quality respectively; Change over time, evidence that attitude measures change or do not change between iterations; ES+/ES++/ES+++, Small, moderate, large effect size respectively; Relationships with other measures, evidence that attitude measures are correlated or not with other measures; Within/without group difference, comparisons between sample sub‐groups or between samples and others.

#### 4.1.3 Objective 3: Attitudinal differences within groups including UK mental health nurses

Hannigan et al.'s (2000) mental health nurse‐only study reported higher scores on the depersonalization subscale of the Maslach Burnout Inventory, a measure of reported behavioural attitudes towards patients, in those with non‐elderly caseloads, those with no job security, and among male staff. Effect size for all these differences was small, and there was no difference between those reporting an unsupportive versus supportive manager. Other differences related to demographics and experiential characteristics in mixed samples. Jansen et al.'s (2006) international comparative study found UK respondents to judge aggression more negatively on dimensions related to offensiveness (large effect), destructiveness, protectiveness and intrusiveness (small and moderate effect sizes). There is some evidence that attitudinal differences exist at the level of gender, experience and job role, education and previous substance use but these were generally inconsistent (Anderson et al., 2000; Jansen et al., 2006; Richmond & Foster, 2003).

#### 4.1.4 Objective 4: Attitudinal change

There was ample evidence from studies that measured attitudes were amenable to change over the short term in relation to personality disorder (Acford & Davies, 2019; Davies et al., 2014; Dickens et al., 2018; Ebrahim et al., 2016; Lamph et al., 2018), self‐harm antipathy (Patterson et al., 2007a,2007b) and mental disorder/substance misuse co‐morbidity (Munro & Baker, 2007; Munro et al., 2007). Effect sizes for pre‐test to post‐test change were typically large except that for the Self‐Harm Antipathy Scale which was moderate. However, sustained change was far less well evidenced across all relevant studies because retention rates at later follow‐up points were generally poor rendering significant results based on completer analysis highly susceptible to bias. Elsewhere, when attitudinal measures were secondary outcomes in trials (e.g., Bowers, Brennan, et al., 2006; Bowers et al., 2015), there was no significant change despite significant improvements in primary outcomes including measures of conflict and containment.

#### 4.1.5 Objective 5: Links between different attitudes or between attitudes and other constructs

The MBI depersonalization subscale was negatively correlated with tolerance of aggression (Whittington, 2002; large effect size), though this relationship was not detected in a different sample (Whittington & Higgins, 2002). Hannigan et al. (2000) found depersonalization to be linked to measures of general health (small effect), self‐esteem, coping and stress (all moderate effect size), and Laker et al. (2019) reported an association with a lack of self‐reported efficacy to effect change. Hosie and Dickens (2018) reported correlations between scales of an amended Self‐Harm Antipathy Scale and their Attitudes to Self‐cutting Management (ASc‐ME) scale suggesting a relationship between lower antipathy and approval of supportive and harm‐reducing self‐harm management techniques (providing first aid kit, giving advice, remaining present during a cutting episode and providing sterile cutting implements). Finally, Bowers, Whittington, et al. (2006) reported significant correlations between subscales of the Attitudes to Personality Disorder Questionnaire (APDQ) and data‐derived factors relating to ward conflict (associated with low “security,” security provision (low APDQ “acceptance” and observation (low “purpose” and low “enthusiasm”).

### 4.2 Practice‐oriented attitudes

Practice‐oriented attitudes were measured in 13 unique samples (see Table 2). Internal reliability was usually supported from the sample or prior research.

#### 4.2.1 Objective 1: Positive, negative and polarized appraisals

Three attitudinal targets were rated at least one standard deviation below the scale midpoint (See Figure 3), these were the measures “refusing treatment to a person who has self‐harmed” and “giving inappropriate treatment to a person who has self‐injured” on the ASc‐ME scale (Hosie & Dickens, 2018) and “mechanical restraint” on the Attitudes to Containment Measures Questionnaire (ACMQ; Bowers et al., 2004). Targets rated by nurses at least one standard deviation above scale midpoint were eight items of the ASc‐ME including “care planning,” “suggest distraction techniques,” “providing advice on wound care,” “provide first aid kit” and “offer PRN.” For general containment, IM medication, constant observation and manual restraint all achieved high ratings in two studies each (Pettit et al., 2016; Whittington et al., 2009) while seclusion was rated so highly only in the latter study. Elsewhere, mental health nurses rated towards the positive end of the scale in relation to neuroleptic treatment (Harris et al., 2007), three aspects of physical health care for people with mental illness (Robson & Haddad, 2012) and ECT (Wood et al., 2007). Attitudinal targets with wide standard deviations suggesting greater range of orientation were those related to physical deterioration in the context of the management of medical emergency in mental health settings (Lavelle et al., 2017).

#### 4.2.2 Objective 2: Attitudinal differences between UK mental health nurses' and other groups

Other results are summarized in Table 3. There were important differences between mental health nurse raters of both the ACMQ and ASc‐ME and patients or previous service users. Whittington et al. (2009) reported that staff respondents had more positive scores than service user respondents on all methods of containment except for open‐area seclusion, mechanical restraint and net bed (effect sizes varies from small for mechanical restraint to large for IM medication). For ratings of management techniques for self‐cutting, nurses rated nine of seventeen techniques more positively than prior users of services, mostly the least restrictive ones but also seclusion and physical restraint (effect sizes ranged from small to large). Other items were rated similarly or, in the case of “refusing treatment,” the effect size was small. Comparisons with other groups showed more positive orientation to mental health nurse prescribing among nurses than psychiatrists (Patel et al., 2009). In Georgieva et al.'s (2020) study of attitudes to mental health legislation, the most important determinant was the country of origin of respondents; only those from the UK and Denmark rated >70% positivity towards their country's relevant law.

#### 4.2.3 Objective 3: Attitudinal differences within groups including UK mental health nurses

Within‐sample analyses revealed that attitudes to containment measures differed across gender; males rated all techniques more positively than female staff except time out, PICU and IM medication, though all effect sizes were small. Age analyses showed younger staff made more positive appraisals of mechanical restraint and net beds; again, effect size was small. Staff who had used specific measures were more positively oriented to them in all cases. Additionally, there was variation by geographic location. While order of positive orientation was similar across three regions, with the sole exception of manual restraint, the level differed by region (small and moderate effect sizes). ACMQ and ASC‐ME relationships reported by Hosie and Dickens (2018) suggested strong but not identical attitudes to similar methods of containment for use in general (ACMQ) and for self‐cutting specifically (ASc‐ME). For the other practice‐related attitudes, within‐group analyses by Harris et al. (2007) suggested there may be professional and experiential associations with attitudes to maintenance neuroleptic treatment where community mental health nurses were significantly more positive than ward‐based mental health nurses; and to physical health care of mental health patient, an additional general nursing qualification was associated with confidence and smoking disapproval (and mental health nurses with past 5‐year training in physical healthcare had a higher total PHASe score than those without.

#### 4.2.4 Objective 4: Attitudinal change

There was little evidence that tested whether practice‐oriented attitudes changed over time; Lavelle et al. (2017) examined change associated with simulation‐based physical healthcare training and reported significant (small effect size) increase medical emergency‐related attitudes in total attitudes score following baseline and intervention.

#### 4.2.5 Objective 5: Links between different attitudes or between attitudes and other constructs

Multilevel modelling did not find ACMQ to be significantly associated with either all self‐harm or moderate self‐harm (Bowers et al., 2008). A data‐derived factor comprising variables related to patient drug/alcohol use and absconding was associated with greater ACMQ acceptability and safety for patients (Bowers, Whittington, et al., 2006).

## 5 DISCUSSION

This review has unified a sizeable but hitherto disparate literature about the measured attitudes of mental health nurses in the UK. Given that attitudes are generally considered to be specific to particular socio‐geographical‐historical contexts then it makes sense to analyse a meaningful subset of contemporary studies, in this case from the UK 2000 to present. While there have been few cross‐cultural studies, it is instructive that investigations into aggression‐related attitudes across Europe (Jansen et al., 2005) and between UK and China (Whittington & Higgins, 2002) provide evidence for such differences. Conceptually, it makes sense to collate empirical literature about mental health nurses' attitudes because the individual studies show them to be perceived to be important, yet up to now studies in relation to particular groups of people or to specific management practices have mostly taken place in isolation from consideration of other attitudes or indeed other constructs. Further reviews of research about specific attitude targets therefore risk compounding the problem of a non‐joined‐up approach. Hence, we made no prior assumptions about which particular attitudinal targets might be related and included all studies irrespective of what, ostensibly, the attitudes investigated were *about*. We found that studies could be classified quite simply in terms of their attitudinal targets, namely as about either specific groups of people or about specific aspects of practice. We had five specific objectives and we discuss each in turn.

### 5.1 Positive, negative and polarized appraisals

We identified studies of mental health nurses' attitudes related to seven types of person‐oriented targets and five types of practice‐oriented targets. Despite the number of relevant studies, there was strong evidence of negatively oriented attitudes, at least in absolute terms, in relation to a limited range of targets. While essentially arbitrary as an indicator, a sample mean and standard deviation entirely below the midpoint indicates below midpoint mean score for around two thirds of the sample providing as good as available an indicator of where priorities for action may lie. This only occurred in the case of borderline personality disorder (negative emotions subscale; Dickens et al., 2018); suicidal behaviour as a “moral evil” (Anderson et al., 2000); substance misuse (permissiveness; treatment intervention orientation and treatment optimism subscales; Richmond & Foster, 2003); and acute mental health (care or control and therapeutic perspective subscales; Baker et al., 2005). Attitudes to personality disorders were also polarized in studies by Markham (2003) and Bowers, Brennan, et al. (2006) and to self‐harm (Patterson et al., 2007a,2007b) suggesting significant numbers with more negatively oriented evaluations. From an evidence‐based perspective, these are the areas that should be prioritized in terms of attitude improvement and in terms of further exploring whether and how attitudes and behaviour are linked phenomena. Importantly, a review of research about the experiences of service users with a diagnosis of borderline personality disorder and their families and carers suggests that they commonly perceived staff in mental health services as judgemental and to hold negative attitudes (Lamont & Dickens, 2019). Similar findings are also prevalent in reviews of studies of the experiences of people who self‐harm (Lindgren et al., 2018) and of people who have spent time in acute mental health care (Schmidt & Uman, 2020). This suggests that, at least in these areas, there is congruence between mental health nurses' measured attitudes and service users' experiences; moreover, that congruence is negative. While these reviews are all international in scope, all of them reference evidence from the UK suggesting that there is little reason to suspect that UK‐based service users' experiences are more positive than anybody else. In relation to people who use mental health services and who hold a comorbid substance misuse diagnosis, similar findings of negative experience are not widespread and it has been suggested that it may be that, in this domain, it is a lack of mental health professional substance use disorder‐specific training that is problematic (Priester et al., 2016).

### 5.2 Attitudinal differences between UK mental health nurses and others

There was little evidence that UK mental health nurses' attitudes differed significantly from other professional or occupational groups. They were more positively oriented to nurse prescribing than psychiatrists (Patel et al., 2009) though this may say more about psychiatrists than nurses. Mental health practitioners including nurses were less negatively oriented to self‐harm behaviour than a sample of general health qualified practitioners (Patterson et al., 2007a,2007b), which has a positive side but does not negate the apparently polarized attitudes of mental health practitioners in the same study and discussed above. UK mental health nurses were less positively oriented to aspects of patient aggression including offensiveness, destructiveness protectiveness and intrusiveness compared with most other European countries' nurses (Jansen et al., 2006). Research from outside of the UK but using the same Attitudes Towards Aggression Scale has suggested that mental health nurses measured attitudes to aggression are largely personal and idiosyncratic rather than clustered by demographic characteristics or by ward (Laiho et al., 2014). The authors suggest as a result that interventions to change attitudes to aggression at, for example, ward level through a culture alteration programme is likely to be less useful than interventions that target the individual. However, UK nurses' attitudes were not alarming in themselves and the practical import of differences between nations is unknown given a lack of studies examining relationships between these attitudes and actual practice.

Of greater salience was findings about the differences between mental health nurses' attitudes and those of people who use or have used services. In relation to the use both of containment measures (Whittington et al., 2009) and measures to manage self‐cutting (Hosie & Dickens, 2018), nurses were significantly more positive than service users for most types of intervention. In the case of containment measures, it is possible that dissonances between attitudes about practices such as seclusion and physical restraint whereby staff are more positively oriented than patients could lead the former to underestimate the negative effect of use of that intervention on the latter. This suggests there is a place in staff aggression management training programmes for education about differences in the relative attitudes; however, we did not locate any research about this specifically (see attitudinal change below). In relation to self‐cutting management, nurses were more positive than service users about most of the least restrictive methods as well as about seclusion and physical restraint. With regard to the former, it may be that nurses are overly expectant about the effectiveness of those interventions. Interestingly, there was no significant discrepancy between nurses and service users in their attitudes to harm reduction strategies including practices such as provision of sterile blades and supporting people while cutting. Further, these strategies ranked similarly in a hierarchy of interventions and were rated by both groups of respondents more positively than seclusion and restraint. Additionally, seclusion and restraint were rated considerably less favourably in Hosie and Dickens (2018) study in relation to self‐cutting management than in Whittington et al.'s (2009) study of their use more generally on a somewhat similarly scaled instrument. This suggests that attitudes to certain containment measures may well differ dependent upon the reason for their use, for example to manage person‐directed violence than to stop self‐harm. This suggests that prevention and management of aggression and violence programmes need to help participants to consider alternative approaches when the aim is to prevent self‐harm and, further, that the appropriate use of harm reduction strategies be considered.

### 5.3 Attitudinal differences within groups that include UK mental health nurses

Bowers, Whittington, et al. (2006) finding that males were more positive in their appraisals of most containment measures than females including seclusion and restraint, and Hannigan et al.'s (2000) that more depersonalized attitudes were found among male mental health staff might suggest that differential approaches to training and support are appropriate. This suggestion is, however, limited by a lack of evidence (see attitudinal change below) of the efficacy of interventions for changing attitudes. However, associational studies (Harris et al., 2007; Robson & Haddad, 2012) finding more positive attitudes to neuroleptic medications in community‐based nurses than ward‐based nurses and to physical health care of people with mental health problems in mental health nurses with an additional adult nursing qualification do suggest that experience and education may have knock‐on benefits for attitudes. Accordingly, career‐long continuing professional development is appropriate.

### 5.4 Attitudinal change

Most research examining change over time in the context of interventions has been limited by a failure to retain participants beyond the immediate end of intervention assessment and thus demonstrate that any change is sustained. This was the case for intervention targeted at borderline personality disorder‐related attitudes (Acford & Davies, 2019; Davies et al., 2014; Dickens et al., 2018; Ebrahim et al., 2016; Lamph et al., 2018), self‐harm antipathy (Patterson et al., 2007b), medical emergency‐related attitudes (Lavelle et al., 2017) and schizophrenia‐related attitudes (Bradshaw et al., 2007). Interestingly, in Bowers, Brennan, et al. (2006), Bowers et al.'s (2015), Safewards studies there was no change in measured personality disorder‐related attitudes over time despite more tangible study outcomes related to reduced occurrences of conflict and containment. We do, however, query whether the tool used in these studies is actually an attitudinal measure (see “relationship between attitudes and between attitudes and other constructs” below). These findings suggest that evaluation studies where a target outcome is attitudinal need to be more rigorously conducted to demonstrate effectiveness beyond the immediate post‐intervention measurement. Further, given Bowers, Brennan, et al. (2006), Bowers et al. (2015) findings that the mechanisms and relationships between education and attitude change need considerable further study to determine whether such interventions are even likely to be useful.

### 5.5 Relationship between attitudes and between attitudes and other constructs

There has been limited research into this aspect. Specifically, personality disorder‐related attitudes were found not to be related to ward‐level self‐harm rates in Bowers et al.'s (2008) highly powered study across 136 acute mental health wards. While this might superficially suggest that attitude‐practice links are not worth pursuing in future research studies, we note that the finding was only true in relation to the APDQ, a measure which, unusually for an attitude scale, captures respondent reports of the frequency of their affinity with items rather than their degree of positive/negative evaluation. Hence, had we been rather stricter in our inclusion criteria, studies using this tool would have been ineligible. Attitudes to Containment Measures Questionnaire scores were also found not to be associated with self‐harm rates in Bowers et al.'s (2008) study, again suggesting that attitudes may not be worth further investigation. However, this may not be the case since the ACMQ is used to investigate measures for disturbed behaviour in particular and not for self‐harm specifically. Further investigations are therefore required to determine whether attitudes to managing self‐harm are associated with its incidence. However, this also speaks to the question of whether extinction of self‐harming behaviour rather than harm reduction should be the measure of success of any practice innovation (James et al., 2017). Further, research is required into differences between attitudes to managing self‐harm specifically with coercive measures and whether these differ from their use for violent and aggressive behaviour. It may be that attitudes other than those more relevant to personality disorder or containment measures play a role in self‐harm rates and these should be investigated more thoroughly. As examples, items contributing to relevant scales on the Attitudes To Acute Mental Health Scale (ATAMHS; Baker et al., 2005) “care or control” (sample item “members of society are at risk from the mentally ill”) and “therapeutic perspective” (“psychiatric patients are generally difficult to like”) do not ostensibly sound compatible with contemporary notions of recovery‐oriented care.

Finally, attitudes as behavioural manifestations of self‐reported depersonalized responses to service users were associated with poorer general health, self‐esteem, stress (Hannigan et al., 2000) and reduced self‐efficacy to effect change (Laker et al., 2019). There were contradictory findings about whether they were associated with tolerance of aggression (Whittington, 2002; Whittington & Higgins, 2002). The former findings suggest that interventions to change attitudes might have associated benefits in the domains of stress and self‐esteem or indeed vice versa. There is some evidence that mindfulness‐based approaches are effective in reducing stress in mental health professionals (Rudaz et al., 2017).

## 6 STRENGTHS AND LIMITATIONS

This review has used a number of techniques which add new insight to the collection of work both in terms of the relative import of the various studies and the unified body as a whole. First, extraction and standardization of means from different measurement tools allow a clear picture to be drawn of what the literature tells us about the measured attitudes of UK mental health nurses. Second, the calculation of standardized effect sizes where possible for all correlations or differences also informs interpretation of the import and relevance of individual studies. Third, the quality assessment of studies has facilitated the weighting of evidence. Fourth, and finally, the integration of these study findings in itself highlights the hitherto lack of such an integrated approach. The review highlights that studies have not examined relationships between attitudes and practice. Key contributions of this review, therefore, are the highlighting of the lack of connectedness between investigation of different attitudes and the lack of studies which take the next step and examine relationships between attitudes and behaviour and/or practice. In short, mental health nursing research has fallen behind contemporary theoretical developments in attitudinal research such as the causal attitude network model (Dalege et al., 2016) which conceptualize clusters of similar attitude types and provide testable models of those structures thus having the potential to inform causal attributions. One immediate implication of this is that new mental health nursing research about attitudes needs to consider using designs based on more contemporary theoretical approaches.

This review concentrates solely on one socio‐geographical‐historical subsample of available attitudinal research, namely that conducted in the UK and from the past 20 years only. Further, studies rarely report mental health nurse data separately from those of other healthcare professionals yet we have included studies which contain only a proportion of mental health nurses. We have concentrated on studies where attitudes are measured using scales. As a result, we excluded a number of studies where questionnaires were administered but results were reported on an item‐by‐item basis. Similarly, we have not included data from qualitative studies. It may be possible for future reviews to widen the socio‐geographical‐historical scope and the type of studies included. Finally, we only included studies of constructs that met our operational definition of “attitude” and other constructs such as “beliefs,” “opinions,” “perceptions” and “ideologies” also warrant attention.

## 7 CONCLUSION

Attitudinal research in UK mental health nursing has proliferated in the last two decades. There is some good evidence that many nurses make negative attitudinal appraisals about personality disorder and substance misuse. However, the extent and importance of this are somewhat shrouded by a lack of connectedness in current approaches to mental health nursing attitudinal research. What is now needed is a focus on a more integrated approach to study using new and innovative techniques based on contemporary models of attitudes.

## 8 RELEVANCE STATEMENT

Attitudes are considered integral to good mental health nursing practice. There has been considerable research about them but the work is disparate and lacks a joined‐up approach. The current paper takes a broader approach and examines the attitudinal literature as a body of work rather than looking at single attitudes. In doing so it highlights where the evidence lies in terms of priorities for future work and identifies the need for new approaches that consider the interconnectedness of attitudes and the links between attitudes and practice.

## ETHICS STATEMENT

The study is a review of existing literature and did not require ethics review.

## Supporting information

Appendix S1

Appendix S2

## Data Availability

The data that support the findings of this study are available from the corresponding author upon reasonable request.

## References

[jpm12826-bib-0001] Acford, E. , & Davies, J. (2019). Exploring therapeutic engagement with individuals with a diagnosis of personality disorder in acute psychiatric inpatient settings: A nursing team perspective. International Journal of Mental Health Nursing, 28, 1176–1185. 10.1111/inm.12629 31286646

[jpm12826-bib-0002] Ajzen, I. (1991). The theory of planned behaviour. Organizational Behavior and Human Decision Processes, 50(2), 179–211. 10.1016/0749-5978(91)90020-T

[jpm12826-bib-0003] Albarracin, D. , & Shavitt, S. (2018). Attitudes and attitude change. Annual Review of Psychology, 69, 299–327. 10.1146/annurev-psych-122216-011911 PMC12479097 28841390

[jpm12826-bib-0004] American Psychological Association . (2022). APA dictionary of psychology. Accessed 2/12/2020 at: https://dictionary.apa.org/

[jpm12826-bib-0005] Anderson, M. , Standen, P. , Nazir, S. , & Noon, J. P. (2000). Nurses’ and doctors’ attitudes towards suicidal behaviour in young people. International Journal of Nursing Studies, 37, 1–11. 10.1016/s0020-7489(99)00057-7 10687805

[jpm12826-bib-0006] Baker, J. A. , Richards, D. A. , & Campbell, M. (2005). Nursing attitudes towards acute mental health care: Development of a measurement tool. Journal of Advanced Nursing, 49, 522–529. 10.1111/j.1365-2648.2004.03325.x 15713184

[jpm12826-bib-0007] Bodner, E. , Cohen‐Fridel, S. , & Iancu, I. (2011). Staff attitudes toward patients with borderline personality disorder. Comprehensive Psychiatry, 52, 548–555. 10.1016/j.comppsych.2010.10.004 21130423

[jpm12826-bib-0008] Bodner, E. , Cohen‐Fridel, S. , Mashiah, M. , Segal, M. , Grinshpoon, A. , Fischel, T. , & Lancu, L. (2015). The attitudes of psychiatric hospital staff toward hospitalization and treatment of patients with borderline personality disorder. BMC Psychiatry, 15, 2. 10.1186/s12888-014-0380-y 25609479 PMC4307152

[jpm12826-bib-0078] Bolton, W. , Feigenbaum, J. , Jones, A. , & Woodward, C. (2010). Development of the PD‐KASQ (Personality Disorder – Knowledge, Attitudes and Skills Questionnaire). unpublished manuscript.

[jpm12826-bib-0009] Bowers, L. , & Allan, T. (2006). The attitude to personality disorder questionnaire: Psychometric properties and results. Journal of Personality Disorders, 20(3), 281–293. 10.1521/pedi.2006.20.3.281 16776556

[jpm12826-bib-0010] Bowers, L. , Brennan, G. , Flood, C. , Lipang, M. , & Oladapo, P. (2006). Preliminary outcomes of a trial to reduce conflict and containment on acute psychiatric wards: City Nurses. Journal of Psychiatric and Mental Health Nursing, 13, 165–172. 10.1111/j.1365-2850.2006.00931.x 16608471

[jpm12826-bib-0079] Bowers, L. , Haglund, K. , Muir‐Cochrane, E. , Nijman, H. , Simpson, A. , & Van Der Merwe, M. (2010). Locked doors: a survey of patients, staff and visitors. Journal of Psychiatric and Mental Health Nursing, 17, 873–880. 10.1111/j.1365-2850.2010.01614.x 21078002

[jpm12826-bib-0011] Bowers, L. , James, K. , Quirk, A. , Simpson, A. , SUGAR , Stewart, D. , & Hodsoll, J. (2015). Reducing conflict and containment rates on acute psychiatric wards: The Safewards cluster randomised controlled trial. International Journal of Nursing Studies, 52, 1412–1422. 10.1016/j.ijnurstu.2015.05.001 26166187 PMC4518134

[jpm12826-bib-0012] Bowers, L. , Simpson, A. , Alexander, J. , Ryan, C. , & Carr‐Walker, P. (2004). Cultures of psychiatry and the professional socialization process: The case of containment methods for disturbed patients. Nurse Education Today, 24, 435–442. 10.1016/j.nedt.2004.04.008 15312952

[jpm12826-bib-0013] Bowers, L. , van der Werf, B. , Vokkolainen, A. , Muir‐Cochrane, E. , Allan, T. , & Alexander, J. (2007). International variation in attitudes to containment measures for disturbed psychiatric inpatients. International Journal of Nursing Studies, 44(3), 357–364. 10.1016/j.ijnurstu.2006.01.005 16524581

[jpm12826-bib-0014] Bowers, L. , Whittington, R. , Nolan, P. , Parkin, D. , Curtis, S. , Bhui, K. , Hackney, D. , Allan, T. , & Simpson, A. (2008). Relationship between service ecology, special observation and self‐harm during acute in‐patient care: City‐128 study. British Journal of Psychiatry, 193, 395–401. 10.1192/bjp.bp.107.037721 18978321

[jpm12826-bib-0015] Bowers, L. , Whittington, R. , Nolan, P. , Parkin, D. , Curtis, S. , Bhui, K. , Hackney, D. , Allan, T. , Simpson, A. , & Flood, C. (2006). The city 128 study of observation and outcomes on acute psychiatric wards. Research report produced for the National Co‐ordinating Centre for the National Institute for Health Research Service Delivery and Organisation Programme (NCCSDO). Retrieved from: https://njl‐admin.nihr.ac.uk/document/download/2008731

[jpm12826-bib-0016] Bradshaw, T. , Butterworth, A. , & Mairs, H. (2007). Does structured clinical supervision during psychosocial intervention education enhance outcome for mental health nurses and the service users they work with? Journal of Psychiatric and Mental Health Nursing, 14, 4–12. 10.1111/j.1365-2850.2007.01021.x 17244000

[jpm12826-bib-0017] Cartwright, A. K. J. (1980). The attitudes of helping agents towards the alcoholic client: The influence of experience, support, training, and self‐esteem. British Journal of Addiction, 75, 413–431. 10.1111/j.1360-0443.1980.tb01406.x

[jpm12826-bib-0018] Chambers, M. , Guise, V. , Vȁlimȁki, M. , Botelho, M. A. B. , Scott, A. , Staniulienē, V. , & Zanotti, R. (2010). Nurses’ attitudes to mental illness: a comparison of a sample of nurses from five European countries. International Journal of Nursing Studies, 47(3), 350–362. 10.1016/j.ijnurstu.2009.08.008 19804882

[jpm12826-bib-0019] Chappel, J. N. , Veach, T. J. , & Krug, R. S. (1985). The substance abuse attitude survey: An instrument for measuring attitudes. Journal of Studies on Alcohol, 46(1), 48–52. 10.15288/jsa.1985.46.48 3974235

[jpm12826-bib-0020] Dagnan, D. , Trower, P. , & Smith, R. (1998). Care staff responses to people with learning disabilities and challenging behaviour: A cognitive‐ emotional analysis. British Journal of Clinical Psychology, 37, 59–68. 10.1111/j.2044-8260.1998.tb01279.x 9547960

[jpm12826-bib-0021] Dalege, J. , Borsboom, D. , van Harreveld, F. , van den Berg, H. , Conner, M. , & van der Maas, H. L. J. (2016). Toward a formalized account of attitudes: The Causal Attitude Network (CAN) model. Psychological Review, 123, 2–22. 10.1037/a0039802 26479706

[jpm12826-bib-0022] Davies, J. , Sampson, M. , Beesley, F. , Smith, D. , & Baldwin, V. (2014). An evaluation of knowledge and understanding framework personality disorder awareness training: Can a co‐production model be effective in a local NHS mental health trust? Personality and Mental Health, 8, 161–168. 10.1002/pmh.1257 24574103

[jpm12826-bib-0023] Dickens, G. L. , Lamont, E. , Mullen, N. , MacArthur, N. , & Stirling, F. (2018). Mixed‐methods evaluation of an educational intervention to change mental health nurses’ attitudes to people diagnosed with borderline personality disorder. Journal of Clinical Nursing, 28(13–14), 2613–2623. 10.1111/jocn.14847 30830704

[jpm12826-bib-0024] Ebrahim, S. , Robinson, S. , Crooks, S. , Harenwall, S. , & Forsyth, A. (2016). Evaluation of awareness level knowledge and understanding framework personality disorder training with mental health staff: impact on attitudes and clinical practice. Journal of Mental Health Training, Education and Practice, 11, 133–143. 10.1108/JMHTEP-07-2015-0030

[jpm12826-bib-0025] Georgieva, I. , Whittington, R. , Lauvrud, C. , Steinert, T. , Wikman, S. , Lepping, P. , Duxbury, J. , Snorrason, J. , Mihai, A. , Berring, L. L. , Bn, R. , & Vesselinov, R. (2020). International variations in mental‐health law regulating involuntary commitment of psychiatric patients as measured by the Mental Health Legislation Attitudes Scale. Medicine, Science and the Law, 59, 104–114. 10.1177/0025802419841139 30982427

[jpm12826-bib-0026] Goffman, E. (1963). Stigma: Notes on the Management of Spoiled Identity. Prentice Hall.

[jpm12826-bib-0027] Greenhalgh, T. (2006). How to read a paper: The basics of evidence based medicine. Blackwell.

[jpm12826-bib-0028] Guise, V. , Chambers, M. , Välimäki, M. , & Makkonen, P. (2010). A mixed‐mode approach to data collection: Combining web and paper questionnaires to examine nurses’ attitudes to mental illness. Journal of Advanced Nursing, 66, 1623–1632. 10.1111/j.1365-2648.2010.05357.x 20497273

[jpm12826-bib-0029] Hannigan, B. , Edwards, D. , Coyle, D. , Fothergill, A. , & Burnard, P. (2000). Burnout in community mental health nurses: findings from the all‐Wales stress study. Journal of Psychiatric and Mental Health Nursing, 7, 127–134. 10.1046/j.1365-2850.2000.00279.x 11146908

[jpm12826-bib-0030] Harris, N. , Lovell, K. , & Day, J. C. (2007). Mental health practitioner’s attitude towards maintenance neuroleptic treatment for people with schizophrenia. Journal of Psychiatric and Mental Health Nursing, 14, 113–119. 10.1111/j.1365-2850.2007.01050.x 17352772

[jpm12826-bib-0080] Herron, J. , Ticehurst, H. , Appleby, J. , Perry, A. , & Cordingley, L. (2001). Attitudes toward suicide prevention in front‐line health staff. Suicide and Life Threatening Behavior, 31, 342–347. 10.1521/suli.31.3.342.24252 11577918

[jpm12826-bib-0031] Hosie, L. , & Dickens, G. L. (2018). Harm‐reduction approaches for self‐cutting in inpatient mental health settings: Development and preliminary validation of the Attitudes to Self‐cutting Management (ASc‐Me) Scale. Journal of Psychiatric and Mental Health Nursing, 25(9–10), 531–545. 10.1111/jpm.12498 30256488

[jpm12826-bib-0032] Howard, L. , & Gamble, C. (2011). Supporting mental health nurses to address the physical health needs of people with serious mental illness in acute inpatient care settings. Journal of Psychiatric and Mental Health Nursing, 18, 105–112. 10.1111/j.1365-2850.2010.01642.x 21299722

[jpm12826-bib-0033] Ingamells, S. , Goodwin, A. M. , & John, C. (1996). The influence of psychiatric hospital and community residence labels on social rejection of the mentally ill. British Journal of Clinical Psychology, 35, 359–367. 10.1111/j.2044-8260.1996.tb01190.x 8889077

[jpm12826-bib-0034] James, K. , Samuels, I. , Moran, P. , & Stewart, D. (2017). Harm reduction as a strategy for supporting people who self‐harm on mental health wards: the views and experiences of practitioners. Journal of Affective Disorders, 214, 67–73. 10.1016/j.jad.2017.03.002 28284098

[jpm12826-bib-0035] Jansen, G. J. , Dassen, T. W. N. , Burgerhof, J. G. M. , & Middel, B. (2005). Psychiatric nurses’ attitudes towards inpatient aggression: preliminary report of the development of attitude towards aggression scale (ATAS). Aggressive Behavior, 32(1), 44–53. 10.1002/ab.20106

[jpm12826-bib-0036] Jansen, G. , Dassen, T. , & Moorer, P. (1997). The perception of aggression. Scandinavian Journal of Caring Science, 11, 51–55. 10.1111/j.1471-6712.1997.tb00430.x 9275822

[jpm12826-bib-0037] Jansen, G. J. , Middel, B. , Dassen, W. N. , & Reijneveld, S. A. (2006). Cross‐cultural differences in psychiatric nurses’ attitudes to inpatient aggression. Archives of Psychiatric Nursing, 20, 82–93. 10.1016/j.apnu.2005.08.012 16549245

[jpm12826-bib-0081] Laiho, T. , Lindberg, N. , Joffe, G. , Putkonen, H. , Hottinen, A. , Kontio, R. , & Sailas, E. (2014). Psychiatric staff on the wards does not share attitudes on aggression. International Journal of Mental Health Systems, 8, 14. 10.1186/1752-4458-8-14 24778708 PMC4002577

[jpm12826-bib-0038] Laker, C. , Matteo, C. , Callard, F. , & Wykes, T. (2019). Why is change a challenge in acute mental health wards? A cross‐sectional investigation of the relationships between burnout, occupational status and nurses’ perceptions of barriers to change. International Journal of Mental Health Nursing, 28, 190–198. 10.1111/inm.12517 29993168 PMC7328713

[jpm12826-bib-0039] Lamont, E. , & Dickens, G. L. (2019). Mental health services, care provision, and professional support for people diagnosed with borderline personality disorder: systematic review of service‐user, family, and carer perspectives. Journal of Mental Health, 17, 1–15. 10.1080/09638237.2019.1608923 31099717

[jpm12826-bib-0040] Lamph, G. , Sampson, M. , Smith, D. , Williamson, G. , & Guyers, M. (2018). Can an interactive e‐learning training package improve the understanding of personality disorder within mental health professionals? Journal of Mental Health Training, Education and Practice, 13, 124–134. 10.1108/jmhtep-03-2017-0023

[jpm12826-bib-0041] Lavelle, M. , Attoe, C. , Tritschler, C. , & Cross, S. (2017). Managing medical emergencies in mental health settings using an interprofessional in‐situ simulation training programme: A mixed methods evaluation study. Nurse Education Today, 59, 103–109. 10.1016/j.nedt.2017.09.009 28968516

[jpm12826-bib-0042] Lindgren, B.‐M. , Svedin, G. S. , & Werko, S. (2018). A systematic literature review of experiences of professional care and support among people who self‐harm. Archives of Suicide Research, 22, 173–192. 10.1080/13811118.2017.1319309 28426393

[jpm12826-bib-0043] Link, B. G. (1987). Understanding labeling effects in the area of mental disorders: An assessment of the effects of expectations of rejection. American Sociological Review, 52, 96–112. 10.2307/2095395

[jpm12826-bib-0044] Link, B. G. , Cullen, F. T. , Frank, J. , & Wozniak, J. F. (1987). The social rejection of former mental patients: Understanding why labels matter. American Journal of Sociology, 92, 1461–1500. 10.1086/228672

[jpm12826-bib-0045] Link, B. G. , & Phelan, J. C. (2001). Conceptualizing stigma. Annual Review of Sociology, 27, 363–385. 10.1146/annurev.soc.27.1.363

[jpm12826-bib-0076] Lipsey, M.W. , & Wilson, D. B. (2001). Practical Meta‐analysis. Sage.

[jpm12826-bib-0046] Maio, G. R. , Haddock, G. , & Verplanken, B. (2018). The psychology of attitudes and attitude change (3rd ed.). Sage Publishing.

[jpm12826-bib-0047] Markham, D. (2003). Attitudes towards patients with a diagnosis of ‘borderline personality disorder’: Social rejection and dangerousness. Journal of Mental Health, 12, 595–612. 10.1080/09638230310001627955

[jpm12826-bib-0048] Maslach, C. , Jackson, S. E. , & Leiter, M. P. (1996). Maslach burnout inventory manual (3rd ed.). Consulting Psychologists Press.

[jpm12826-bib-0049] Maslach, C. , Jackson, S. E. , & Leiter, M. P. (1997). Maslach burnout inventory: third edition. In C. P. Zalaquet , & R. J. Wood (Eds.), Evaluating stress: A book of resources (pp. 191–218). Scarecrow Education.

[jpm12826-bib-0050] Mistral, W. , Hall, A. , & McKee, P. (2002). Using therapeutic community principles to improve the functioning of a high care psychiatric ward in the UK. International Journal of Mental Health Nursing, 11, 10–17. 10.1046/j.1440-0979.2002.00220.x 12400102

[jpm12826-bib-0051] Moher, D. , Liberati, A. , Tetzlaff, J. , & Altman, D. G. , PRISMA Group (2009). Preferred reporting items for systematic reviews and meta‐analyses: the PRISMA statement. PLoS Medicine, 6(7), e1000097. 10.1371/journal.pmed.1000097 19621072 PMC2707599

[jpm12826-bib-0052] Morris, R. , Scott, P. A. , Cocoman, A. , Chambers, M. , Guise, V. , Välimäki, M. , & Clinton, G. (2011). Is the community attitudes towards the mentally ill scale valid for use in the investigation of European nurses’ attitudes towards the mentally ill? A confirmatory factor analytic approach. Journal of Advanced Nursing, 68, 460–470. 10.1111/j.1365-2648.2011.05739.x 21679227

[jpm12826-bib-0054] Munro, S. , & Baker, J. A. (2007). Surveying the attitudes of acute mental health nurses. Journal of Psychiatric and Mental Health Nursing, 14, 196–202. 10.1111/j.1365-2850.2007.01063.x 17352783

[jpm12826-bib-0053] Munro, A. , Watson, H. E. , & McFadyen, A. (2007). Assessing the impact of training on mental health nurses’ therapeutic attitudes and knowledge about co‐morbidity: A randomised controlled trial. International Journal of Nursing Studies, 44, 1430–1438. 10.1016/j.ijnurstu.2006.07.024 16996517

[jpm12826-bib-0055] Patel, M. X. , deZoysa, N. , Baker, D. , & David, A. S. (2005). Depot antipsychotic medication and attitudes of community psychiatric nurses. Journal of Psychiatric and Mental Health Nursing, 12, 237–244. 10.1111/j.1365-2850.2004.00826.x 15788043

[jpm12826-bib-0056] Patel, M. X. , Robson, D. , Rance, J. , Ramirex, N. M. , Memon, T. C. , Bressington, D. , & Gray, R. (2009). Attitudes regarding mental health nurse prescribing among psychiatrists and nurses: A cross‐sectional questionnaire study. International Journal of Nursing Studies, 46, 1467–1474. 10.1016/j.ijnurstu.2009.04.010 19482282

[jpm12826-bib-0057] Patel, M. X. , Yeung, F. K. K. , Haddad, P. M. , & David, A. S. (2008). Psychiatric nurses’ attitudes to antipsychotic depots in Hong Kong and comparison with London. Journal of Psychiatric and Mental Health Nursing, 15, 758–766. 10.1111/j.1365-2850.2008.01306.x 18844802

[jpm12826-bib-0058] Patterson, P. , Whittington, R. , & Bogg, J. (2007a). Testing the effectiveness of an educational intervention aimed at changing attitudes to self‐harm. Journal of Psychiatric and Mental Health Nursing, 14, 100–105. 10.1111/j.1365-2850.2007.01052.x 17244012

[jpm12826-bib-0059] Patterson, P. , Whittington, R. , & Bogg, J. (2007b). Measuring nurse attitudes towards deliberate self‐harm: The Self‐Harm Antipathy Scale (SHAS). Journal of Psychiatric and Mental Health Nursing, 14, 438–445. 10.1111/j.1365-2850.2007.01102.x 17635251

[jpm12826-bib-0060] Pettit, S. A. , Bowers, L. , Tulloch, A. , Biggin Moylan, L. , Sethi, F. , McCrone, P. , Baker, J. , Quirk, A. , & Stewart, D. (2016). Acceptability and use of coercive methods across differing service configurations with and without seclusion and/or psychiatric intensive care units. Journal of Advanced Nursing, 73, 966–976. 10.1111/jan.13197 27809370 PMC5347866

[jpm12826-bib-0061] Priester, M. A. , Browne, T. , Iachini, A. , Clone, S. , DeHart, D. , & Seay, K. D. (2016). Treatment access barriers and disparities among individuals with co‐occurring mental health and substance use disorders: An integrative literature review. Journal of Substance Abuse Treatment, 61, 47–59. 10.1016/j.jsat.2015.09.006 26531892 PMC4695242

[jpm12826-bib-0062] Richmond, I. C. , & Foster, J. H. (2003). Negative attitudes towards people with co‐morbid mental health and substance misuse problems: An investigation of mental health professionals. Journal of Mental Health, 12, 393–403. 10.1080/0963823031000153439

[jpm12826-bib-0063] Robson, D. , & Haddad, M. (2012). Mental health nurses’ attitudes towards the physical health care of people with severe and enduring mental illness: The development of a measurement tool. International Journal Nursing Studies, 49, 72–83. 10.1016/j.ijnurstu.2011.07.011 21899840

[jpm12826-bib-0064] Robson, D. , Haddad, M. , Gray, R. , & Gournay, K. (2013). Mental health nursing and physical health care: A cross‐sectional study of nurses’ attitudes, practice, and perceived training needs for the physical health care of people with severe mental illness. International Journal of Mental Health Nursing, 22, 409–417. 10.1111/j.1447-0349.2012.00883.x 23066812

[jpm12826-bib-0065] Rogers, M. , Wattis, J. , Stephenson, J. , Khan, W. , & Curran, C. (2019). A questionnaire‐based study of attitudes to spirituality in mental health practitioners and the relevance of the concept of spiritually competent care. International Journal of Mental Health Nursing, 28, 1165–1175. 10.1111/inm.1262 31286640

[jpm12826-bib-0066] Rudaz, M. , Twohig, M. P. , Ong, C. W. , & Levin, M. E. (2017). Mindfulness and acceptance‐based trainings for fostering self‐care and reducing stress in mental health professionals: A systematic review. Journal of Contextual Behavioral Science, 6, 380–390. 10.1016/j.jcbs.2017.10.001

[jpm12826-bib-0067] Sandford, D. M. , Kirtley, O. J. , Lafit, G. , Thwaites, R. , & O'Connor, R. C. (2020). An investigation into the factor structure of the attitudes to suicide prevention scale. Crisis, 41, 97–104. 10.1027/0227-5910/a000608 31310166

[jpm12826-bib-0068] Schmidt, M. , & Uman, T. (2020). Experiences of acute care by persons with mental health problems: An integrative literature review. Journal of Psychiatric and Mental Health Nursing, 27, 789–806. 10.1111/jpm.12624 32083776

[jpm12826-bib-0069] Shaw, J. , Minoudis, P. , Hamilton, V. , & Craissati, J. (2012). An investigation into competency for working with personality disorder and team climate in the probation service. Probation Journal, 59, 39–48.

[jpm12826-bib-0070] Taylor, S. M. , & Dear, M. J. (1981). Scaling community attitudes toward the mentally ill. Schizophrenia Bulletin, 7, 225–240. 10.1093/schbul/7.2.225 7280561

[jpm12826-bib-0071] University of York Centre for Reviews and Dissemination (2008). Systematic reviews: CRD's guidance for undertaking reviews in health care. Available at: https://www.york.ac.uk/media/crd/Systematic_Reviews.pdf

[jpm12826-bib-0072] Watson, H. E. , Maclaren, W. , Shaw, F. , & Nolan, A. (2003). Measuring staff attitudes to people with drug problems. Effective Interventions Unit, Scottish Executive.

[jpm12826-bib-0073] Whittington, R. (2002). Attitudes toward patient aggression amongst mental health nurses in the ‘zero tolerance’ era: associations with burnout and length of experience. Journal of Clinical Nursing, 11, 819–825. 10.1046/j.1365-2702.2002.00659.x 12427188

[jpm12826-bib-0074] Whittington, R. , Bowers, L. , Nolan, P. , Simpson, A. , & Neil, L. (2009). Approval ratings of inpatient coercive interventions in a national sample of mental health service users and staff in England. Psychiatric Services, 60, 792–798. 10.1176/ps.2009.60.6.792 19487349

[jpm12826-bib-0075] Whittington, R. , & Higgins, L. (2002). More than zero tolerance? Burnout and tolerance for patient aggression amongst mental health nurses in China and the UK. Acta Psychiatrica Scandinavica, 106(Suppl. 412), 37–40. 10.1034/j.1600-0447.106.s412.8.x 12072124

[jpm12826-bib-0077] Wood, J. H. , Chambers, M. , & White, S. J. (2007). Nurses’ knowledge of and attitude to electroconvulsive therapy. Journal of ECT, 23, 251–254. 10.1097/yct.0b013e31813e0692 18090698

