# Supplementary material for: Mental health nurses' measured attitudes to people and practice: Systematic review of UK empirical research 2000–2019
Source: J Psychiatr Ment Health Nurs. 2022 Feb 23;29(6):788–812. doi: 10.1111/jpm.12826 (PMC9790366; doi:10.1111/jpm.12826)
Supplement: Supplementary file 1 — Appendix S1 [file JPM-29-788-s002.docx]

| **Appendix 1: Study quality assessment** | | | | | | | | | | | | | | | | |
| --- | --- | --- | --- | --- | --- | --- | --- | --- | --- | --- | --- | --- | --- | --- | --- | --- |
|  | | **Aims** | **Sampling** | | | | | **Questionnaire development** | | | | | **General** | | **Overall** | |
| **Study and scale combination** | | **Explicit aims (re: attitudes)** | **Sample size justification** | **Well described sample** | **Representative sample** | **Explicit inclusion/exclusion criteria** | **Response rate % (>50%= 1)** | **Questionnaire development described** | **Questionnaire wording available** | **Internal reliability** | **Test retest reliability** | **External validity** | **Discussion of generealisability** | **Funding statement** | **Total/12** | **Overall quality (0-4 L, 5-8 M, 9-12 H)** |
|  |  |  |  |  |  |  |  |  |  |  |  |  |  |  |  |  |
|  |  |  |  |  |  |  |  |  |  |  |  |  |  |  |  |  |
| Acford & Davies (2019) | PD-KASQ | 0 | 0 | 1 | U | 1 | U | 1 | 1 | 1 | 0 | 0 | 1 | 0 | 5 | M |
| Anderson et al. (2000) | SOQ | 1 | 0 | 1 | U | 1 | 55.9 | 1 | 1 | 0 | 1 | 0 | 1 | 0 | 7 | M |
| Baker et al. (2005) | AT-AMHS | 1 | 0 | 1 | U | 1 | 56 | 1 | 1 | 1 | 0 | 0 | 1 | 0 | 7 | M |
| Bowers et al. (2006a) | APDQ | 1 | 0 | 0 | U | 0 | U | 1 | 1 | 1 | 1 | 0 | 0 | 1 | 5 | M |
|  | MBI (DP) | 1 | 0 | 0 | U | 0 | U | 1 | 1 | 1 | 1 | 1 | 0 | 1 | 6 | M |
| Bowers 2006b 2008; Whittington et al. 2009 | APDQ | 1 | 0 | 1 | U | 1 | U | 1 | 1 | 0 | 0 | 0 | 1 | 1 | 6 | M |
|  | ACMQ | 1 | 0 | 1 | U | 1 | U | 1 | 1 | 0 | 0 | 0 | 1 | 1 | 6 | M |
| Bowers et al. (2010) | ACMQ Locked Doors parallel version | 0 | 0 | 1 | U | U | 52 | 1 | 1 | 0 | 0 | 0 | 0 | 1 | 4 | L |
| Bowers et al. (2015) | SHAS | 0 | 1 | 1 | U | 1 | 61.4 | 1 | 1 | 1 | 0 | 0 | 1 | 1 | 8 | M |
| Bowers et al. (2015) | APDQ | 0 | 0 | 1 | U | 1 | 61.4 | 1 | 1 | 1 | 1 | 0 | 0 | 1 | 7 | M |
| Bradshaw et al. (2007) | Attitudes & Assumptions MCQ | 1 | 0 | 1 | U | 1 | 100 | 1 | 1 | 0 | 0 | 0 | 1 | 0 | 6 | M |
| Davies et al. (2014) | PD-KASQ | 0 | 0 | 0 | U | 0 | U | 1 | 1 | 1 | 0 | 0 | 1 | 0 | 3 | L |
| Dickens et al. (2018) | BPD EAI | 1 | 0 | 1 | U | 1 | 100 | 1 | 1 | 1 | 0 | 0 | 1 | 1 | 8 | M |
|  | BPD CAI | 1 | 0 | 1 | U | 1 | 100 | 1 | 1 | 1 | 0 | 0 | 1 | 1 | 8 | M |
| Ebrahim et al. (2016) | PD-KASQ | 1 | 0 | 0 | U | 0 | 92.3 | 1 | 1 | 1 | 0 | 0 | 1 | 0 | 5 | M |
| Georgieva et al. (2019) | MHLAS | 1 | 0 | 0 | U | 1 | U | 1 | 1 | 1 | 0 | 0 | 1 | 1 | 6 | M |
| Guise et al. (2010) | CAMI | 0 | 0 | 1 | U | 1 | 56.1 | 1 | 1 | 1 | 0 | 1 | 1 | 1 | 8 | M |
| Hannigan et al. (2000) | MBI (DP) | 1 | 1 | 1 | U | 1 | 49 | 1 | 1 | 1 | 1 | 1 | 1 | 0 | 9 | H |
| Harris et al. (2007) | SANTI | 1 | 0 | 1 | U | 1 | U | 1 | 1 | 1 | 1 | 1 | 1 | 0 | 8 | M |
| Hosie & Dickens (2018) | Asc-Me | 1 | 1 | 1 | U | 1 | U | 1 | 1 | 1 | 1 | 1 | 1 | 1 | 10 | H |
|  | SHAS | 1 | 1 | 1 | U | 1 | U | 1 | 1 | 1 | 1 | 1 | 1 | 1 | 10 | H |
|  | ACMQ | 1 | 1 | 1 | U | 1 | U | 1 | 1 | 0 | 1 | 0 | 1 | 1 | 8 | M |
| Jansen et al. (2005, 2006) | ATAS | 1 | 0 | 1 | U | 0 | U | 1 | 1 | 1 | 0 | 0 | 1 | 0 | 5 | M |

|  | | **Aims** | **Sampling** | | | | | **Questionnaire development** | | | | | **General** | | **Overall** | |
| --- | --- | --- | --- | --- | --- | --- | --- | --- | --- | --- | --- | --- | --- | --- | --- | --- |
| **Study and scale combimation** | | **Explicit aims (re: attitudes)** | **Sample size justification** | **Well described sample** | **Representative sample** | **Explicit inclusion/exclusion criteria** | **Response rate % (>50%= 1)** | **Questionnaire development described** | **Questionnaire wording available** | **Internal reliability** | **Test retest reliability** | **External validity** | **Discussion of generealisability** | **Funding statement** | **Total/12** | **Overall quality (0-4 L, 5-8 M, 9-12 H)** |
|  |  |  |  |  |  |  |  |  |  |  |  |  |  |  |  |  |
|  |  |  |  |  |  |  |  |  |  |  |  |  |  |  |  |  |
| Laker et al. (2019) | MBI (DP) | 1 | 1 | 1 | U | 1 | 81.2 | 1 | 1 | 1 | 1 | 1 | 1 | 0 | 10 | H |
| Lamph et al. (2017) | PD-KASQ | 1 | 0 | 0 | U | 0 | 100 | 1 | 1 | 1 | 0 | 0 | 1 | 0 | 5 | M |
| Lavelle et al. (2017) | Purpose built | 1 | 0 | 1 | U | 1 | 100 | 1 | 1 | 0 | 0 | 0 | 1 | 1 | 7 | M |
| Markham (2003) | Social distance | 1 | 0 | 1 | U | 1 | U | 1 | 1 | 1 | 0 | 0 | 1 | 0 | 7 | M |
|  | Beliefs about dangerousness | 1 | 0 | 1 | U | 1 | U | 1 | 1 | 1 | 0 | 0 | 1 | 0 | 7 | M |
|  | Treatment optimism | 1 | 0 | 1 | U | 1 | U | 1 | 1 | 0 | 0 | 0 | 1 | 0 | 6 | M |
| Mistral et al (2002) | Attitudes Measure' | 1 | 0 | 0 | U | 1 | U | 1 | 1 | 0 | 0 | 0 | 0 | 1 | 5 | M |
| Morris et al. (2011) | CAMI | 1 | 0 | 1 | U | 1 | 32.4 | 1 | 1 | 1 | 0 | 0 | 1 | 1 | 7 | M |
| Munro et al. (2007) | CMPPQ | 1 | 1 | 1 | U | 1 | 79.6 | 1 | 1 | 1 | 0 | 0 | 1 | 1 | 9 | H |
| Patel et al. (2005, 2008) | Purpose built | 1 | 0 | 1 | U | 1 | 67 | 1 | 1 | 0 | 1 | 0 | 1 | 0 | 7 | M |
| Patel et al. (2009) | Purpose built | 1 | 0 | 1 | U | 1 | 45 | 1 | 1 | 1 | 0 | 0 | 1 | 1 | 7 | M |
| Patterson et al. (2007ab) | SHAS | 1 | 0 | 0 | U | 1 | U | 1 | 1 | 1 | 0 | 0 | 1 | 0 | 5 | M |
| Pettit et al (2016) | ACMQ | 1 | 1 | 1 | U | 1 | U | 1 | 1 | 0 | 0 | 0 | 1 | 1 | 8 | M |
| Richmond & Foster (2003) | SAAS | 1 | 0 | 1 | U | 1 | 57 | 1 | 1 | 1 | 0 | 0 | 1 | 0 | 7 | M |
| Robson & Haddad (2012, 2013) | PHASe | 1 | 1 | 1 | U | 1 | 52 | 1 | 1 | 1 | 0 | 0 | 1 | 1 | 9 | H |
| Rogers et al. (2019) | Purpose built | 1 | 0 | 1 | U | 1 | U | 1 | 1 | 1 | 0 | 0 | 1 | 1 | 7 | M |
| Sandford et al. (2019) | ASPS | 1 | 1 | 0 | U | 1 | 25 | 1 | 1 | 1 | 0 | 0 | 1 | 1 | 7 | M |
| Whittington (2002) | POAS tolerance subscale | 1 | 0 | 1 | U | 1 | 37 | 1 | 1 | 1 | 0 | 0 | 1 | 0 | 6 | M |
|  | MBI (DP) | 1 | 0 | 1 | U | 1 | 37 | 1 | 1 | 1 | 1 | 1 | 1 | 0 | 8 | M |
| Whittington & Higgins (2002) | POAS tolerance subscale | 1 | 0 | 0 | U | 0 | U | 1 | 1 | 1 | 0 | 0 | 1 | 0 | 4 | L |
|  | MBI (DP) | 1 | 1 | 0 | U | 0 | U | 1 | 1 | 1 | 1 | 1 | 1 | 0 | 8 | M |
| Wood et al. (2007) | Purpose built | 1,Y | 0 | 1 | U | 0 | 38 | 1 | 1 | 1 | 0 | 0 | 1 | 0 | 5 | M |
| 1 = Meets criteria 0 = does not meet criteria U = Unclear. For details of individual scales see Table 3 & 4 | | | | | | | | | | | | | | | | |
