# Supplementary material for: Mental health nurses' measured attitudes to people and practice: Systematic review of UK empirical research 2000–2019
Source: J Psychiatr Ment Health Nurs. 2022 Feb 23;29(6):788–812. doi: 10.1111/jpm.12826 (PMC9790366; doi:10.1111/jpm.12826)
Supplement: Supplementary file 2 — Appendix S2 [file JPM-29-788-s001.docx]

**Appendix 2: Specific attitudinal scales searched**

- Affect Scale/ Affective Reaction Scale (AS / ARS) - Penn (0)
- Alcohol Problems Perceptions Questionnaire (AAPPQ) - Shaw 1978 (2)
- Attitudes to Containment Measures Questionnaire (ACMQ) - Bowers (25)
- Attitude to Personality Disorder Questionnaire (APDQ) - Bowers 2002 (15)
- Attitudes Towards Acute Mental Health Scale (ATAMHS / AT-AMHS) - Baker et al. 2005 (5)
- Attitude to Mental illness Questionnaire (AMIQ) - Luty based on Cunningham (4)
- Attitude Towards Aggression Scale (ATAS), (4)
- Attitudes towards mental health problems scale (ATMHPS) - (2) Unknown originalauthors not named in references list in Master et al (2016). Attitudes towards mental health problems scale (ATMHPS): Confirmatory factor analysis and validation in the Portuguese population. American Journal of Psychiatric Rehabilitation, 19(3), 206-222.
- Attitudes Towards Physical Assault Questionnaire (Poster and Ryan, 1989) (4)
- Attitudes to Self-cutting Management scale (ASc-Me) - Hosie& Dickens (1)
- Attitudes to Suicide Prevention scale (ASPS) - Herron et al., 2001) (5)
- Attribution Questionnaire (AQ) - Corrigan (2)
- Beliefs About Mental Health Nursing An Ideology Scale - Unknown (5)
- Borderline Personality Disorder - Cognitive Attitudes Inventory (BPD CAI) - Bodner et al (1)
- Borderline Personality Disorder - Emotional Attitudes Inventory (BPD EAI) - Bodner et al (1)
- Causal Beliefs Questionnaire (0) - not called this but used in Carter et al 2017 and based on Lobban, F., Barrowclough, C., & Jones, S. (2005). Assessing cognitive representations of mental health problems: I. The illness perception questionnaire for schizophrenia. British Journal of Clinical Psychology, 44, 147–162.
- Community Attitudes towards the Mentally Ill scale (CAMI) (Taylor et al. 1979, Taylor & Dear 1981) (11)
- Comorbidity Problems Perceptions Questionnaire (CMPPQ) based on SAAS as per Munro (0)
- Drug Attitiude Scale (DAS) - Parker (29)
- Management of Aggression and Violence Attitude Scale (MAVAS) Duxbury( 2002) (22)
- Mental Illness Clinicians’ Attitudes Scale (MICA v2) Kassam et al (2010)
- Mental Illness Clinicians’ Attitudes Scale (MICA v4) Gabbidon et al (2013) (5 for any version)
- Mental Health Knowledge Schedule (MHKS) (9) - Evans-Lacko S, Little K, Meltzer H, Rose D, Rhydderch D, Henderson C, et al. (2010). Development and psychometric properties of the Mental Health Knowledge Schedule. Can J Psychiatry; 55: 440–8.
- Mental Health Legislation Attitudes Scale (MHLAS) - Georgieva et al (2019) (1)
- Opinion about Mental Illness Scale (OMI) - Cohen &Struening (29)
- Perceived Devaluation and Discrimination Scale (PDD) - Pattyn et al 2014 (4)
- Perception of Aggression Scale (POAS) both this and ATAS mentioned in Jansen whose is it? (14)
- Physical Healthcare Attitudes Scale (PHASe) - Robson & Haddad Physical Health Attitude Scale (11)
- Prejudice towards People with Mental Illness (PPMI)- Kenny, A., Bizumic, B., & Griffiths, K. M. (2018). The: structure and validity of the Prejudice towards People with Mental Illness (PPMI). BMC psychiatry, 18(1), 293. (61)
- Psychiatric Disability Attribution Questionnaire (PDAQ) - Corrigan et al (2003) (21)
- Questionnaire on Opinions about mental illness (QO) - Magliano et al (1999) (1)
- Recovery Attitude Questionnaire (RAQ) - Borkin JR, Steffen JJ, Ensfield LB, et al. Recovery attitudes questionnaire: development and evaluation. Psychiatric Rehabilitation Journal. 2000;24(2):95–102. (14)
- Recovery Knowledge Inventory (RKI) - Bedregal LE, O’Connell M, Davidson L. The recovery knowledge inventory: assessment of mental health staff knowledge and attitudes about recovery. Psychiatric Rehabilitation Journal. 2006;30(2):96–103. (17)
- Recovery Promoting Relationship Scale (RPRS) - Russinova Z, Rogers SE, Ellison ML. Recovery Promoting Relationship Scale, Centre of Rehabilitation Sciences. Boston, Mass, USA: Boston University; 2006. (4)
- Self-Harm Antipathy Scale (SHAS) - Patterson (13)
- Short Alcohol and Alcohol Problems Perception Questionnaire (SAAPPQ) - Cartwright A, Shaw S, Spratley T. Designing a comprehensive community response to problems of alcohol abuse. London: Department of Health and Social Security, 1975. [25] Anderson P, Clement S. The AAPPQ Revisited: the measurement of general practitioners’ attitudes to alcohol problems. Addiction 1987;82:753–9. (4)
- Social Distance Scale (SDS) - Bogardus (1926) Bogardus Social Distance Scale (BSDS). Also versions by Whatley, Penn, Link, Phillips (5)
- Staff Attitude to Neuroleptic Treatment Inventory (SANTI) Harris et al (2007) (6)
- Substance Abuse Attitude Survey (SAAS) - Chappel (12)
- Suicide Opinion Questionnaire (SOQ) - Domino et al 1982 (22)
- Views of the Therapeutic Environment (VOTE) - Laker (10)
- Mental Health Beliefs and Literacy Scale (2)
- Multidimensional attitude toward mental health scale (1)
- Attitudes Toward Mental Health Treatment Scale (ATMHT). The ATMHT is comprised of 20 items with a four-point Likert scale, and is intended to reflect an individual's attitude toward professional mental health treatment. (1)
- The ATMHT is a modified version of the 29-item Attitudes Toward Seeking Professional Psychological Help Scale (ATSPPHS) (Fisher and Turner 1970). (11)
- Marlowe-Crowne Social Desirability Scale
- Mental Illness Beliefs Inventory (MIBY) - Loureiro et al., 2006) (3)
- The Stigma of Suicide Scale (SOSS (5)
- Expectations for the Employability of People with Serious Mental Illness Scale.
- Personality Disorder-Knowledge, Attitudes and Skills Questionnaire (PD-KASQ) (Bolton et al., 2010).
- Nurses Attitudes towards Obesity and Obese Patients Scale
- Families Importance in Nursing Care: Nurses' Attitudes Scale (FINC-NA-R)Family Nurse Practice Scale (FNPS) Family Nurse Practice Scale (FNPS) (Hsiao & Tsai, [**2015**](https://onlinelibrary-wiley-com.ezproxy.uws.edu.au/doi/full/10.1111/jan.14049#jan14049-bib-0024); Simpson & Tarrant, [**2006**](https://onlinelibrary-wiley-com.ezproxy.uws.edu.au/doi/full/10.1111/jan.14049#jan14049-bib-0043)).
- Families’ Importance in Nursing Care: Nurses’ Attitudes (FINC‐NA) (Benzein, Johansson, Arestedt, Berg, Johansson, Arestedt, Berg, & Saveman, [**2008**](https://onlinelibrary-wiley-com.ezproxy.uws.edu.au/doi/full/10.1111/jan.14049#jan14049-bib-0008); Blöndal et al., [**2014**](https://onlinelibrary-wiley-com.ezproxy.uws.edu.au/doi/full/10.1111/jan.14049#jan14049-bib-0010); Hsiao & Tsai, [**2015**](https://onlinelibrary-wiley-com.ezproxy.uws.edu.au/doi/full/10.1111/jan.14049#jan14049-bib-0024); Luttik et al., [**2017**](https://onlinelibrary-wiley-com.ezproxy.uws.edu.au/doi/full/10.1111/jan.14049#jan14049-bib-0028); Pascual Fernández et al., [**2015**](https://onlinelibrary-wiley-com.ezproxy.uws.edu.au/doi/full/10.1111/jan.14049#jan14049-bib-0039)) and three further versions of the scale: a revised version (FINC‐NA‐R) (Gusdal, Josefsson, Thors Adolfsson, & Martin, [**2017**](https://onlinelibrary-wiley-com.ezproxy.uws.edu.au/doi/full/10.1111/jan.14049#jan14049-bib-0021); Linnarsson, Benzein, & Årestedt, [**2015**](https://onlinelibrary-wiley-com.ezproxy.uws.edu.au/doi/full/10.1111/jan.14049#jan14049-bib-0027); Saveman, Benzein, Engström, &Årestedt, [**2011**](https://onlinelibrary-wiley-com.ezproxy.uws.edu.au/doi/full/10.1111/jan.14049#jan14049-bib-0041)), a short version (FINC‐NA‐S) (Mackie, Marshall, Mitchell, & Ireland, [**2017**](https://onlinelibrary-wiley-com.ezproxy.uws.edu.au/doi/full/10.1111/jan.14049#jan14049-bib-0029)) and a modified Portuguese version (IFCE‐AE) (Oliveira et al.., [**2011**](https://onlinelibrary-wiley-com.ezproxy.uws.edu.au/doi/full/10.1111/jan.14049#jan14049-bib-0038)).
- Family Nurse Caring Belief Scale (FNCBS) (Meiers, Tomlinson, &Peden‐McAlpin, [**2007**](https://onlinelibrary-wiley-com.ezproxy.uws.edu.au/doi/full/10.1111/jan.14049#jan14049-bib-0032)).
- Family‐Centered Care Questionnaire (FCCQ) (Bruce & Ritchie, [**1997**](https://onlinelibrary-wiley-com.ezproxy.uws.edu.au/doi/full/10.1111/jan.14049#jan14049-bib-0012)) and a revised version (FCCQ‐R) (Alabdulaziz, Moss, & Copnell, [**2017**](https://onlinelibrary-wiley-com.ezproxy.uws.edu.au/doi/full/10.1111/jan.14049#jan14049-bib-0001); Bruce et al.,[**2002**](https://onlinelibrary-wiley-com.ezproxy.uws.edu.au/doi/full/10.1111/jan.14049#jan14049-bib-0011); Caty, Larocque, &Koren,[**2001**](https://onlinelibrary-wiley-com.ezproxy.uws.edu.au/doi/full/10.1111/jan.14049#jan14049-bib-0014); Coyne, Murphy, Costello, O'Neill, & Donnellan, [**2013**](https://onlinelibrary-wiley-com.ezproxy.uws.edu.au/doi/full/10.1111/jan.14049#jan14049-bib-0016))
- Measure of Beliefs about Participation in Family‐Centered Service (MBP‐FCS) (Asai, [**2011**](https://onlinelibrary-wiley-com.ezproxy.uws.edu.au/doi/full/10.1111/jan.14049#jan14049-bib-0004); King et al., [**2003**](https://onlinelibrary-wiley-com.ezproxy.uws.edu.au/doi/full/10.1111/jan.14049#jan14049-bib-0025)).
- Evidence-based Practice Attitude Scale-36 (EBPAS-36) Norwegian version.  (used in Attitudes of mental health providers towards adoption of evidence-based interventions: relationship to workplace, staff roles and social and psychological factors at work). Marte Rye, Oddgeir Friborg & Ingunn Skre
- Attitudes towards recovery scale (ARQ)
- Evidence-Based Practices Attitudes Scale and the Modified Practice Attitude Scale
- [**Attitude Towards Deliberate Self-Harm Questionnaire**](https://www-scopus-com.ezproxy.uws.edu.au/record/display.uri?eid=2-s2.0-84938557162&origin=resultslist&sort=plf-f&src=s&nlo=&nlr=&nls=&sid=2d0bbdd070ffa45a9d72fa18e971e0ab&sot=a&sdt=a&sl=194&s=ABS%28%22Attitude%22%29+AND+ABS+%28%22mental+health+nurse%22+OR+%22psychiatric+nurse%22%29+AND+PUBYEAR+%3e+1999+AND+ALL%28%22united+kingdom%22+OR+%22England%22+OR+%22Scotland%22+OR+%22wales%22+OR+%22northern+ireland%22+OR+%22great+britain%22%29&relpos=91&citeCnt=0&searchTerm=)
